# Alkyl-Substituted δ-Lactones Derived from Dihydrojasmone and Their Stereoselective Fungi-Mediated Conversion: Production of New Antifeedant Agents

**DOI:** 10.3390/molecules21091226

**Published:** 2016-09-13

**Authors:** Anna Gliszczyńska, Damian Semba, Maryla Szczepanik, Katarzyna Dancewicz, Beata Gabryś

**Affiliations:** 1 Department of Chemistry, Wroclaw University of Environmental and Life Sciences, Norwida 25, Wrocław 50-375, Poland; 2 Bioprocess and Biomedical Engineering Division, Wroclaw University of Technology, Norwida 4/6, Wrocław 50-373, Poland; damiansmb@gmail.com; 3 Department of Invertebrate Zoology, Nicolaus Copernicus University, Lwowska 1, Toruń 87-100, Poland; mszczep@umk.pl; 4 Department of Botany and Ecology, University of Zielona Góra, Szafrana 1, Zielona Góra 65-516, Poland; k.dancewicz@wnb.uz.zgora.pl (K.D.); b.gabrys@wnb.uz.zgora.pl (B.G.)

**Keywords:** dihydrojasmone, alkyl-substituted δ-lactones, oxyfunctionalization, Baeyer–Villiger oxidation

## Abstract

A chemoenzymatic method was applied to obtain optically pure alkyl-substituted δ-lactones. First, chemical Baeyer–Villiger oxidation of dihydrojasmone (**1**) was carried out, affording two new alkyl-substituted δ-lactones: 3,4-dihydro-5-methyl-6-pentyl-2*H*-pyran-2-one (**2**) and 5-methyl-6-pentyl-1,13-dioxabicyclo[4.1.0]heptan-2-one (**3**). In the next step, fungal strains were investigated as biocatalysts to enantioselective conversion of δ-lactones (**2**) and (**3**). The fungal cultures: *Fusarium culmorum* AM10, *Fusarium equiseti* AM15 and *Beauveria bassiana* AM278 catalyzed the stereoselective hydration of the double bond of lactone (**2**) (ee = 20%–99%) while *Didymosphaeria igniaria* KCh6670 proved to be the best biocatalyst for the reduction of carbonyl group in the epoxylactone (**3**) (ee = 99%). In both cases, chiral oxyderivatives were obtained in low to high yields (7%–91%). The synthetic lactones (**2**), (**3**) and its derivatives (**4**), (**5**) were tested for their antifeedant activity towards larvae and adults of lesser mealworm (*Alphitobius diaperinus* Panzer) and peach potato aphid (*Myzus persicae* [Sulzer]) and some of them were active towards studied insects.

## 1. Introduction

Over the last few decades, numerous research papers about isolation and synthesis of alkyl-substituted δ-lactones have been widely published in the literature [1,2,3]. These compounds have been isolated from various natural sources: plants, marine organisms and terrestrial animals [4,5,6]. Because of their manifold biological properties, they are of marked interest not only from a chemical, but also from a practical point of view. Chiral alkyl-substituted δ-lactones as the carriers of specific odor have considerable industrial value in the flavor, cosmetic and food industries. They are also a large group of compounds that play important roles in insect’s lives as pheromones, and are the key intermediates for the synthesis of other biologically active compounds. δ-Decalactone has been reported by Lopez and Morgan as a component of the warning odor of North America porcupine *Erethizon dorsatum* [7]. That compound has been also detected in venom-producing mandibular glands of ponerine ant *Pachycondyla apicalis* [8]. A chiral building block, which is a synthetically versatile precursor and has attracted attention over the years, is a parasorbic acid. Its (+)-enantiomer isolated from mountain ash berries (*Sorbus aucuparia*) is an intermediate for the synthesis of *cis*-3,6-dimethyltetrahydropyran-2-one, the major component of the male carpenter bee pheromone [9]. The component of defence secretion of two species of formicin ants of the genus *Camponotus* [6] is another naturally occurring alkyl-substituted δ-lactone, (*R*)-(−)-massoialactone isolated from the bark oil of *Cryptocarya massoia* (Lauraceae) [10] and jasmine flowers and leaves of *Polianthes tuberosa* L. [11]. Goniothalamin, a bioactive styryl lactone, isolated from the leaves of *Goniothalamus* species [12] possesses insecticidal activity against beet armyworm *Spodoptera exigua* Hübner (Lepidoptera: Noctuidae) [13]. This compound increases mortality of beet armyworm by reducing food consumption, moulting inhibition and damage to the gut. Goniothalamin is also highly effective against larvae of the mosquito, *Culex quinquefasciatus* Say [14], and possesses valuable medicinal properties [15,16].

In our research project, we are exploring new compounds that can be useful for insect control. Based on the literature data, we identified alkyl-substituted δ-lactones as an aim of our next studies. Searching the appropriate substrate for their synthesis, we focused our attention on the recent findings in research of jasmonates (JAs). The various JAs are described as compounds that allow plants to respond specifically to alterations in the environment and play the role of hormones [17,18,19]. We selected dihydrojasmone (**1**) as a substrate for the synthesis of alkyl-substituted δ-lactones. The chemoenzymatic pathway of novel jasmonate signaling compounds generated in this study was initiated with the Baeyer–Villiger oxidation of dihydrojasmone (**1**) to alkyl-substituted δ-lactones (**2**) and (**3**). Biological properties of lactones are often strongly related with occurrence in their structure, the additional oxygen function, and the absolute configuration of chiral centers. Therefore, looking for stereoselective methods of synthesis of optically pure oxyderivatives of lactone (**2**) and (**3**), we applied microbial transformations as the method from the field of green chemistry, which possesses overwhelming advantages over chemical synthesis, and we employed the wild fungal strains from our collection as biocatalysts. In the present work, we also describe the results of evaluation of biological activity of obtained lactones (**2**), (**3**) and their enantiomerically enriched oxyderivatives (**4**), (**5**) towards larvae and adults of lesser mealworm (*Alphitobius diaperinus* Panzer) and the peach potato aphid (*Myzus persicae* [Sulzer]) and discuss their possible modes of action.

## 2. Results and Discussion

### 2.1. Chemical Oxidation of Dihydrojasmone *(**1**)*

**Figure 1:**
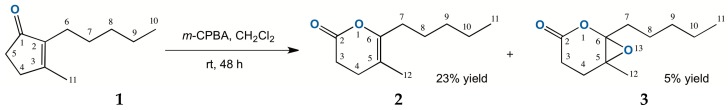
Oxidation of dihydrojasmone (**1**) with *m*-CPBA.

Taking into consideration the broad spectrum of alkyl-substituted δ-lactones properties, it was purposeful to check whether lactone moiety introduced into the structure of dihydrojasmone (**1**) indicate the antifeedant activity. The Baeyer–Villiger oxidation of commercially available dihydrojasmone (**1**) with *meta*-chloroperbenzoic acid (*m*-CPBA) generated the mixture of two new δ-lactones: 3,4-dihydro-5-methyl-6-pentyl-2*H*-pyran-2-one (**2**) and 5-methyl-6-pentyl-1,13-dioxabicyclo[4.1.0]heptan-2-one (**3**) as the products (Figure 1) that were separated by silica-gel column chromatography. These products were obtained in 23% and 5% yields, respectively. Their structures were confirmed by spectral data (Figures S1–S14). Relatively low yields can be caused by the formation of further oxidation products in the reaction with highly reactive *m*-CPBA. The possible product could also be diol formed by the opening of a oxirane ring, which, due to its high polarity, was not isolated from the reaction mixture or chromatographic separation.

The absorption bands at 1761 cm^−1^ (**2**) and 1743 cm^−1^ (**3**) in the IR spectra indicate the presence of the δ-lactone ring in these molecules. The value of chemical shifts of carbonyl carbon atom C-2 (210.13 ppm and 212.13 ppm for lactones (**2**) and (**3**), respectively) found in the ^13^C-NMR spectra are characteristic for disubstituted γ,δ-enol lactones, which was reported by Mandal and Jawalkar [20]. The place of insertion of oxygen atoms to ketone rings was determined on the basis signals from carbon atoms C-6 of lactones (**2**) and (**3**) in ^13^C-NMR spectra, which were shifted from 170.0 ppm (C-2 in substrate (**1**)) to 175.19 ppm (lactone **2**) and 67.44 ppm (lactone **3**). These facts clearly proved that oxygen atoms were inserted into cyclopentenone rings between carbon atoms C-1 and C-2 of dihydrojasmone (**1**). Moreover, the presence in the spectrum of (**2**) signals at ^13^C-NMR confirmed that the double bond in the cyclopentene ring was not affected during the oxidation process. The opposite observation was confirmed for lactone (**3**), where spectral data provide evidence that the oxirane ring was formed in the place of double bond. In the ^13^C-NMR spectrum of lactone (**3**), the signals of atoms C-5 and C-6 (69.51 and 67.44 ppm) undoubtedly confirm the presence of epoxy ring in the structure of product (**3**). The signals found in the ^1^H-NMR spectra also proved the structures of lactones (**2**) and (**3**). The data presented above confirmed the high regioselectivity of chemical Baeyer–Villiger oxidation, and, according to the mechanism of this reaction, the more substituted carbon atom migrated to the electrophilic oxygen atom.

### 2.2. Biotransformation of 3,4-Dihydro-5-methyl-6-pentyl-2H-pyran-2-one *(**2**)*

Our studies concentrated on the evaluation of the usefulness of wild fungal strains in the obtaining of enantiomerically enriched oxyderivatives of alkyl-substituted δ-lactones from the corresponding lactones (**2**) and (**3**). In the first step, we performed small-scale biotransformations, using portions of 10 mg of substrates. The screening experiments were performed using 13 fungal strains of the genus: Absidia, Penicillum, Cunninghamella, Fusarium, Botrytis, Beauveria, Chaetomium, Didymosphaeria, Mortierella and Syncefalastrum. Those biocatalysts were selected based on their proven ability to enantioselective hydroxylation [18,21]. Among the tested fungal strains, three—*Fusarium culmorum* AM10, *Fusarium equiseti* AM15 and *Beauveria bassiana* AM278—were able to catalyze the conversion of lactone (**2**) into the more polar derivative. No formation of any biotransformation products of (**2**) in the culture of another microorganisms was observed even after nine days of incubation. The reaction course was monitored by means of thin layer chromatography (TLC) and gas chromatography (GC). The GC analysis provided the information about changes in quantitative ratio of product in the course of the process. The optical purity of isolated products was determined by GC analysis with application of chiral column (CP-Chirasil-DEX CB).

**Figure 2:**
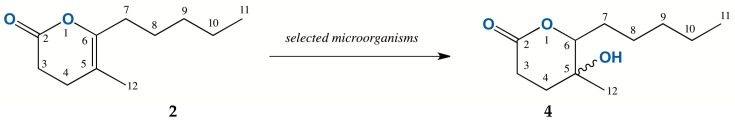
Biotransformation of (**2**) with *F. culmorum* AM10, *F. equiseti* AM15 and *B. bassiana* AM278.

Selected strains of fungi performed regio- and enantioselective hydration of C=C double bond of 3,4-dihydro-5-methyl-6-pentyl-2*H*-pyran-2-one (**2**) to 5-hydroxy-5-methyl-6-pentyltetrahydro-2*H*-pyran-2-one (**4**) (Figure 2).

The structure of (**4**) was established on the basis of its spectral data (Figures S15–S18). The absorption band at 3445 cm^−1^ in the IR spectrum of this product confirms the presence of hydroxy group in the molecule of (**4**), whereas the band at 1765 cm^−1^ proved that the δ-lactone moiety stays untouched. Hydration of the double bond of (**2**) was confirmed by signals in the ^13^C-NMR spectrum of (**4**). The signal from the carbon atom C-6 observed at 89.39 ppm in the spectrum of substrate (**2**) is shifted up to the higher frequency at 76.90 ppm. Additionally, the value of chemical shift of C-5 carbon atom (89.26 ppm in the ^13^C-NMR spectrum) is characteristic for carbon atoms connected with oxygen atoms. The presence of doublet of doublets at δ = 3.46 ppm with the coupling constants *J* = 8.9 and 3.1 Hz from proton H-6 in the ^1^H-NMR spectrum of hydroxylactone (**4**) not visible in the spectrum of substrate indicates the hydration of the double bond in the lactone ring. Singlets from the protons of methyl group at C-5 in the spectrum of product are shifted (1.30 ppm) in comparison with its location in the ^1^H-NMR spectrum of **2** (1.41 ppm). Such a difference in chemical shift of these protons indicates that the methyl group is connected to carbon atoms bonded directly with hydroxy group. The product of biotransformation 5-hydroxy-5-methyl-6-pentyltetrahydropiran-2-one (**4**) is a new compound has not been published before.

**Figure 3:**
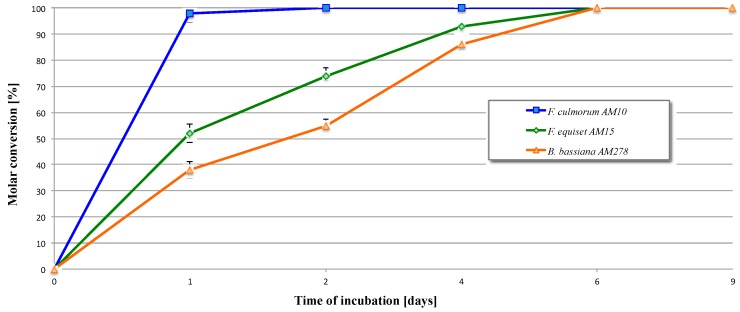
Time course of the transformation of lactone (**2**) by selected fungal strains.

Microbial transformation of δ-lactones (**2**) in the culture of *F. culmorum* AM10 proceeded with high rate (Table 1, entry 1). After 24 h of biotransformation, 98% (according to GC (S23)) of hydroxylactone (**4**) in the reaction mixture was observed (Figure 3). On the next day, the substrate was fully converted and no further oxidation products were detected in the reaction mixture. 5-Hydroxy-5-methyl-6-pentyl-tetrahydropiran-2-one (**4**) was obtained in high yield 91% after one day as (−)-isomer with ee = 20%.

In the case of biotransformation of (**2**) catalyzed by enzymatic system of *F. equiseti* AM15, after the first 24 h, 48% of the unreacted substrate was observed in the reaction mixture (GC) (Figure 3). In the next days of incubation, the reaction rate was reduced and the process of hydration proceeded slower. The amount of substrate (**2**) gradually decreased, whereas the amount of product (**4**) increased proportionally. The complete conversion of (**2**) was achieved after six days (Table 1, entry 2). Product (**4**) was isolated from *F. equiseti* AM15 culture at high yield (53%) in optically pure form as dextrorotatory enantiomer.

As it is presented in Table 1 (entry 3), the reaction rate performed by *B. bassiana* AM278 was lower than in the case of *F. culmorum* AM10 and *F. equiseti* AM15. After one-day incubation of substrate (**2**), the products mixture contained only 38% of product (**4**) (Figure 3). Its amount increased at a low rate during the progress of the biotransformation and after six days reached 100%. *B. bassiana* AM278 turn out to be an effective biocatalyst for the enantiospecific oxidation of δ-lactones (**2**) and produced hydroxylactone (**4**) as pure (+)-enantiomer, although in a much smaller isolated yield of only 7%.

### 2.3. Biotransformation of 5-Methyl-6-pentyl-1,13-dioxabicyclo[4.1.0]heptan-2-one *(**3**)*

**Figure 4:**
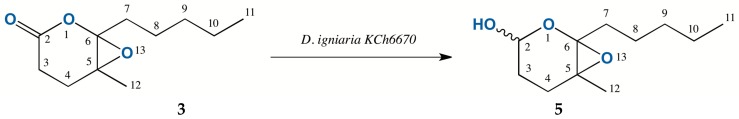
Biotransformation of (**3**) with *D. igniaria* KCh6670.

**Figure 5:**
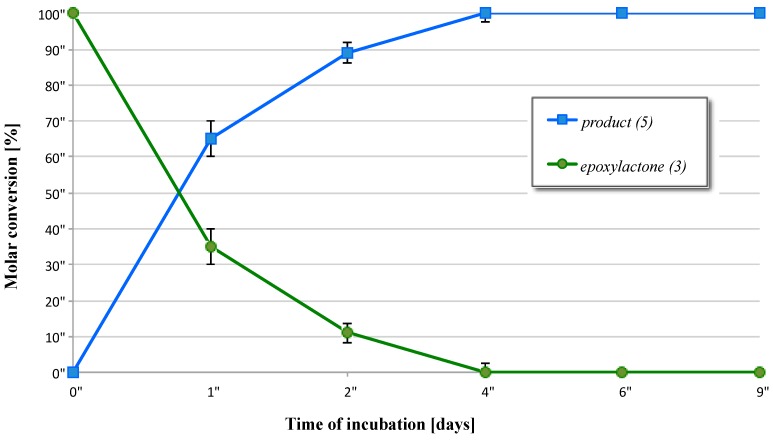
Time dependence of transformation of lactone (**3**) in *D. igniaria* KCh6670 culture.

In the next step of our project, leading to optically active oxygenated derivatives of alkyl-substituted δ-lactones, epoxylactone (**3**) was subject to the biotransformation. Surprising results were found for microbial conversion of 6-methyl-1-pentyl-2,7-dioxabicyclo[4.1.0]heptan-3-one (**3**). Among tested microorganisms, only the culture of *D. igniaria* KCh6670 catalyzed the conversion of lactone (**3**). This time, we observed the enantioselective reduction of carbonyl group (Figure 4). After 24 h of incubation, the conversion of epoxylactone (**3**) reached 65% and systematically increased with time (Figure 5). After four days, epoxylactone (**3**) was completely transformed to the product (**5**). The preparative biotransformation of 5-methyl-6-pentyl-1,13-dioxabicyclo[4.1.0]heptan-2-one (**3**) in the culture of *D. igniaria* KCh6670 gave after four days (+)-5-methyl-6-pentyl-1,13-dioxabicyclo[4.1.0]heptan-2-ol (**5**), which was isolated in 48% yield as single enantiomer (ee = 99%).

The structure of formed 5-methyl-6-pentyl-1,13-dioxabicyclo[4.1.0]heptan-2-ol (**5**) was established by means of ^1^H-NMR and ^13^C-NMR (S19–S22). Reduction of carbonyl group in lactone ring is proved by doublet at 4.31 ppm (*J* = 6.0 Hz) from proton H-2 in the ^1^H-NMR spectrum. The evidence for the presence of the hydroxy group in the structure of product (**5**) provided also ^13^C-NMR spectrum, where the signal of C-2 was moved from δ = 212.13 ppm for substrate (**3**) to δ = 73.52 ppm for the product (**5**). Furthermore, the absorption band at 3430 cm^−1^ in the IR spectrum indicates that bioreduction had taken place.

### 2.4. Feeding Deterrent Activity Against the Lesser Mealworm

In the present studies, the antifeedant activity of the tested compounds varied significantly. The results presented in Table 2 clearly revealed that activity is dependent on the developmental stage. Considering the total coefficients of deterrence, it may be concluded that dihydrojasmone (**1**) was a better deterrent for larvae than adults. A detailed analysis shows, however, that in the choice tests, this compound was a very strong deterrent for both stages, and these differences were minor. The marked differences were observed in no-choice tests. For adults, dihydrojasmone (**1**) was an attractant, whereas for larvae was a moderate antifeedant. The introduction of lactone moiety into the structure of dihydrojasmone (**1**) leads to a change in activity of the resulting lactones. The increase in activity against both stages was observed in the case of the unsaturated δ-lactone (**2**). The particularly strong increase in activity against adults was observed. The weak antifeedant activity, also against both stages, was showed by a second obtained lactone, i.e., saturated bicyclic δ-lactone (**3**). Hydroxylactone (**4**) as pure (−)-enantiomer, obtained by biotransformation of δ-lactone (**2**) in the culture of *F. culmorum* AM10, was a very strong feeding deterrent against larvae and adults of the lesser mealworm. Its (+)-enantiomer produced by *F. equiseti* AM15 was a very strong antifeedant against adults, but its activity against larvae was weak. In the no-choice test, this compound showed attractant properties. The product of biotransformation of bicyclic δ-lactone (**3**) with participation of *D. igniaria* KCh6670 (**5**) was a very good deterrent for larvae, but only in the choice test. In the no-choice situation, its antifeedant properties were very weak.

The comparison of the antifeedant activities of the compounds studied against two developmental stages of the lesser mealworm shows the stronger sensitivity of adults than larvae. The phenomenon of higher sensory sensitivity of adults of *A. diaperinus* to antifeedants in our previous studies was also observed [22].

In light of these results, only δ-hydroxylactone (−)-(**4**), due to the strong inhibition of feeding in both larvae and adults, would be the best candidate for practical use in integrated pest management of *A. diaperinus*. Two very strong antifeedants against adults, i.e., δ-lactone (**2**) and δ-hydroxylactone (+)-(**4**) may also affect the population size. The starvation is also often associated with other biological effects against insects, such as antioviposition and reduction of fertility [23].

### 2.5. Deterrent Activity against the Peach Potato Aphid

Aphids possess the piercing-sucking mouthparts composed of four thin stylets, which determine their mode of feeding. The stylets pierce through the plant surface and probe through outer plant tissues—epidermis and parenchyma—to reach vascular elements from which the phloem sap is ingested [24]. Moreover, aphids do not have external taste chemoreceptors on their mouthparts, so they are not able to distinguish between attractant and deterrent components of their food prior to the insertion of stylets into the plant [25]. Due to these circumstances, the process of aphid feeding is hidden from the human eye and impossible to monitor directly. The monitoring of settling and/or behaviour during settling in a choice-test is therefore the commonly applied method to evaluate aphid responses to various chemicals applied to their otherwise accepted host plants [26]. In the present study, the settling success of the peach potato aphid on plants treated with hydroxyjasmone (**1**), and its derivatives varied depending on the compound applied and the duration of time after exposure (Table 3).

Hydroxyjasmone (**1**) and the unsaturated δ-lactone (**2**) showed weak but not significant deterrent properties while the δ-hydroxylactone (–)-(**4**) was a weak attractant. The activities of hydroxyjasmone (**1**) and δ-hydroxylactone (−)-(**4**) ceased in the course of time while the activity of δ-lactone (**2**) was relatively persistent. The saturated bicyclic δ-lactone (**3**) was significantly highly attractant to the peach potato aphid: the preference of aphids for the treated leaves was revealed as soon as 1 h after treatment and persisted at least for 24 h that was the end of the experiment (Table 3).

The results of the aphid settling choice-test can be supplemented with the results of direct monitoring of aphid behaviour during initial 15 min of contact with the studied compounds (no-choice test). The primarily deterrent and then inactive in the choice settling test, hydroxyjasmone (**1**), evoked relatively negative initial aphid responses: the duration of presence of aphids on treated leaves was significantly shorter and the total probing time when aphids were on a treated leaf was nearly twice as short as on control untreated leaves. As aphids clearly avoided the hydroxyjasmone (**1**)-treated leaves soon after exposure and their probing activity was repressed, hydroxyjasmone (**1**) can be considered as having repellent properties. However, this repellent activity is short-lived: the deterrent effect ceased by the second h after exposure as was shown in the aphid-settling assay (Table 3 and Table 4). Aphid initial behaviour on leaves treated with unsaturated δ-lactone (**2**) and δ-hydroxylactone (−)-(**4**) did not differ significantly from control. The weak deterrent activity of δ-lactone (**2**) and weak attractant activity of δ-hydroxylactone (−)-(**4**), which was found during the settling assay must have resulted from the longer time of aphid exposure to those chemicals. In contrast, on the saturated bicyclic δ-lactone (**3**)-treated leaves, there were less probes and an individual probe was 1.6 times longer than on control leaves. It means that aphids were prevented from the withdrawal of their stylets from the plant tissues by the addition of the δ-lactone (**3**), which confirmed the strong attractant character of this lactone to *M. persicae* that was found during the 24 h settling choice experiment.

A variety of compounds has been studied to find prospective aphid repellents or deterrents, including DEET, the *N*,*N*-diethyl-m-methylbenzamide [27]. Very strong behaviour-modifying activities towards *M. persicae* and other aphid species, e.g., *Acyrthosiphon pisum* and *Rhopalosiphum maidis,* are shown by compounds of terpenoid character and certain plant essential oils that include lower terpenoids, which was demonstrated by various researchers, e.g., [27,28,29,30]. Structural transformations of the original molecule and enantiomeric purity can enhance or change the profile of this activity. For example, the natural compound piperitone appeared rather neutral or weakly deterrent to *M. persicae* and the introduction of a lactone moiety into the molecule caused a significant increase in the deterrent activity [31]. β-Damascone appeared a weak attractant close to not active to *M. persicae*, but modifications of its structure (lactonization, incorporation of halogen atoms) caused the avoidance of treated leaves by aphids during settling and reluctance to probe in choice- and no-choice experiments [26]. In another study, (*R*)-(+)-pulegone appeared a strong deterrent, while its (*S*)-(−)-isomer did not have any effect on the behaviour of *M. persicae* [30].

In plant protection strategies that include the use of semiochemicals, both the attractants and the deterrents may play an important role. The so-called stimulo-deterrent diversionary strategy (=‘push–pull’ system) proposes the use of deterrents to ‘push’ the insect pest away from the crop and attractants to ‘pull’ the pests to non-crop plants away from the protected field [32]. Considering the results of the present experiments, the saturated bicyclic δ-lactone (**3**) with clear attractant properties towards *M. persicae* seems a very prospective candidate for incorporation in integrated aphid control.

## 3. Materials and Methods

### 3.1. General Experimental Procedures

Analytical TLC was performed on aluminium plates coated with silica gel (Kieselgel 60, F_254_, Merck, Darmstadt, Germany) with mixture of hexane, acetone and diethyl ether in various ratios as developing systems. Compounds were detected by spraying the plates with the solution of: Ce(SO_4_)_2_ (1 g), H_3_[P(Mo_3_O_10_)_4_] (2 g) in 10% H_2_SO_4_, followed by heating to 120–200 °C.

Column chromatography was performed on silica gel (Kieselgel 60, 230–400 mesh ASTM, Merck) with mixture of hexane, acetone and diethyl ether in various ratios as eluents.

Gas chromatography (GC) analysis was carried out on an Agilent Technologies 6890N Network GC (Agilent Technologies, Waldbronn, Germany) equipped with flame ionization detector (FID) using H_2_ as the carrier gas and capillary column (HP-5, 30 m × 0.32 mm × 0.25 µm). The temperature program was as follows: injector 250 °C, detector (FID) 250 °C, column temperature: 70 °C, 70–175 °C (rate 20°/min), 175–300 °C (rate 40°/min), 300 °C (hold 2 min); *R*_f_ (**2**) = 0.44, *R*_f_ (**3**) = 0.35, *R*_f_ (**4**) = 0.58, R_f_ (**5**) = 0.29, *R*_f_ (**6**) = 0.50; *t*_R_ (**1**) = 2.8 min, *t*_R_ (**2**) = 3.5 min, *t*_R_ (**3**) = 2.4 min, *t*_R_ (**4**) = 4.2 min, *t*_R_ (**5**) = 3.1 min.

Chiral gas chromatography was carried out on a CP-Chirasil-DEX CB (25 m × 0.25 mm × 0.25 µm) column (Varian/Chrompack, Middelburg, The Netherlands). Enantiomeric excesses were determined with the following temperature program: injector 250 °C, detector (FID) 250 °C, column temperature: 80 °C, 80–200 °C (rate 0.6°/min), 200 °C (hold 1 min); *t*_R_ (+)-(**4**) = 113.7 min, *t*_R_ (−)-(**4**) = 106.9 min, *t*_R_ (+)-(**5**) = 110.4 min.

^1^H-NMR, ^13^C-NMR, DEPT 135, HSQC and ^1^H-^1^H COSY spectra were recorded in CDCl_3_ solutions on a Brüker Avance AMX 300 and 600 spectrometer (Bruker, Rheinstetten, Germany). Chemical shifts were referenced to the residual solvent signal (δ_H_ 7.26, δ_C_ 77.0). IR spectra were determined using Mattson IR 300 Thermo-Nicolet spectrophotometer (Mattson, Warszawa, Poland). Optical rotations were determined on a JASCO P-200-Na polarimeter (Easton, PA, USA) in a version with an iRM controller using dichloromethane as a solvent, concentration denoted in g/100 mL.

High resolution mass spectra (HRMS) were recorded using electron spray ionization (ESI) technique on spectrometer Waters ESI-Q-TOF Premier XE (Waters Corp., Millford, MA, USA).

### 3.2. Chemical Synthesis

The solution of *meta*-chloroperbenzoic acid (*m*-CPBA) (16.17 g, 0.072 mol, 77%, Aldrich, Saint Louis, MO, USA) in dichloromethane (20 mL) was dried over anhydrous MgSO_4_ and then filtered on a funnel. Such prepared solution of peracid was added dropwised to the stirring solution of dihydrojasmone (**1**) (4 g, 0.024 mol) (purchased from Sigma Aldrich) in anhydrous dichloromethane (30 mL) cooled in ice bath. The mixture was then stirred for 48 h at room temperature. The progress of the reaction was monitored with TLC and GC. When the reaction was completed the reaction mixture was washed with sodium sulphite (Na_2_SO_3_) and sodium bicarbonate (NaHCO_3_), brine, dried over anhydrous MgSO_4_ and filtered. The solvent was evaporated in vacuo. The crude mixture of products was subjected to column chromatography. Elution with hexane/ether (2:1) (*v*/*v*) gave lactone (**2**) and epoxylactone (**3**) (23% and 5% yield, respectively).

*3,4-Dihydro-5-methyl-6-pentyl-2H-pyran-2-one* (**2**): Colorless liquid; ^1^H-NMR (CDCl_3_): δ (ppm) 0.88 (t, 3H, *J* = 7.2 Hz, CH_3_-11), 1.30–1.32 (m, 4H, CH_2_-9, CH_2_-10), 1.41 (s, 3H, CH_3_-12), 1.53-1.64 (m, 2H, CH_2_-8), 1.75 (ddd, 1H, , *J* = 13.8, 10.2, 6.0 Hz, one of CH_2_-7), 1.89 (ddd, 1H, *J* = 13.8, 10.2, 5.4 Hz, one of CH_2_-7), 2.09 (ddd, 1H, , *J* = 15.0, 13.2, 5.4 Hz, one of CH_2_-4), 2.23 (ddd, 1H, *J* = 15.0, 7.8, 1.8 Hz, one of CH_2_-4), 2.42 (ddd, 1H, , *J* = 16.8, 5.4, 1.8 Hz, one of CH_2_-3), 2.58 (ddd, *J* = 16.2, 13.2, 7.8 Hz, one of CH_2_-3), ^13^C-NMR (CDCl_3_): δ (ppm) 210.13 (C-2), 175.19 (C-6), 89.99 (C-5), 31.56 (C-9), 31.20 (C-7), 27.61 (C-3), 26.02 (C-4), 23.59 (C-8), 22.50 (C-10), 17.98 (C-12), 13.95 (C-11). IR (film, cm^−1^): 1761 (s), 1154 (m). HRMS: calcd for C_11_H_18_O_2_ [M + H]^+^: 183.1385, found 183.1381.

*5-Methyl-6-pentyl-1,13-dioxabicyclo[4.1.0]heptan-2-one* (**3**): Colorless liquid; ^1^H-NMR (CDCl_3_): δ (ppm) 0.88 (t, 3H, *J* = 6.6 Hz, CH_3_-11), 1.26–1.34 (m, 4H, CH_2_-9, CH_2_-10), 1.41–1.46 (m, 2H, CH_2_-8), 1.51 (s, 3H, CH_3_-12), 1.60 (m, 1H, one of CH_2_-7), 1.77 (m, 1H, one of CH_2_-7), 1.87 (ddd, 1H, *J* = 13.8, 9.6, 8.4 Hz, one of CH_2_-4), 2.04 (ddd, 1H, *J* = 18.0, 9.6, 1.8 Hz, one of CH_2_-3), 2.24 (dd, 1H, *J* = 13.8, 9.6 Hz one of CH_2_-4), 2.32 (ddd, 1H, *J* = 18.0, 9.6, 8.4 Hz, one of CH_2_-3), ^13^C-NMR (CDCl_3_): δ (ppm) 212.13 (C-2), 69.51 (C-5), 67.44 (C-6), 32.09 (C-9), 31.93 (C-3), 27.61 (C-4), 24.62 (C-8), 23.51 (C-7), 22.47 (C-10), 16.58 (C-12), 13.96 (C-11). IR (film, cm^−1^): 1743 (s), 1458 (w), 1056 (w). HRMS: calcd for C_11_H_18_O_3_ [M + Na]^+^: 221.1154, found 221.1157.

### 3.3. Microorganisms

All microorganisms came from the Department of Chemistry Wrocław University of Environmental and Life Sciences, City, Country. The fungi and yeast were grown and maintained on Sabouraud 4% dextrose-agar slopes (Difco, Detroit, MI, USA) at 4 °C and freshly subcultured before use in the transformation experiments. Exponentially growing cells were used as inoculums for all the experiments. The composition of the culture medium was as follows: 3 g glucose; 1 g aminobac; and 100 mL of water.

### 3.4. Screening Procedure

Erlenmayer flasks (300 mL), each containing 100 mL of the sterile medium, were inoculated with a suspension of microorganisms and then incubated for 48–72 h at 25 °C on a rotatory shaker (150 rpm). After full growth of the culture, 10 mg of a substrate dissolved in 1 mL of acetone was added. Control cultivation with no substrate was also performed. To monitor the reactions, after 1, 2, 4, 6 and 9 days, a 5-mL incubation mixture was withdrawn from each flask using a sterile syringe and immediately extracted with dichloromethane (3 × 10 mL). The extracts were dried over MgSO_4_, concentrated in vacuo and analysed by TLC. Quantitative analysis of the mixtures was performed by means of GC. All experiments were carried out in triplicates. Results in Figure 3 are reported as means of triplicate experiments ± standard deviations (SD).

### 3.5. Preparative Biotransformation

A portion of 1 mL of the pre-incubation culture solution were used to inoculate two or three 2000 mL flasks, each containing 500 mL of the cultivation medium. The cultures were inoculated at 25 °C for 48–72 h on a rotary shaker (150 rpm). Then, 50 mg of a substrate dissolved in 5 mL of acetone was added to each flask. After 1–6 days (depends on the culture) of incubation, the mixtures were extracted with dichloromethane (3 × 300 mL), dried (MgSO_4_) and concentrated in vacuo. The transformation products were separated by column chromatography. The yields of the reactions, physical and spectral data of obtained products are given below:

*Biotransformation of (***2***) by* F. culmorum *AM10.* Biotransformation of lactone (**2**) (0.1 g), after one day, gave (−)-(**4**) (ee = 20%) as the only product. After column chromatography, 0.1 g (91% yield) of product (−)-(**4**) was isolated, [α]${}_{D}^{20}$ = −2.11° (*c* = 0.95, CH_2_Cl_2_).

*Biotransformation of (***2***) by* F. equiseti *AM15.* Biotransformation of lactone (**2**) (0.1 g), after six days, gave (+)-(**4**) (ee = 99%) as the only product. After column chromatography, 0.058 g (53% yield) of product (+)-(**4**) was isolated, [α]${}_{D}^{20}$ = +4.80° (*c* = 1.45, CH_2_Cl_2_).

*Biotransformation of (***2***) by* B. bassiana *AM278.* Biotransformation of lactone (**2**) (0.1 g), after six days, gave (+)-(**4**) (ee = 99%) as the only product. After column chromatography, 0.008 g (7% yield) of product (+)-(**4**) was isolated, [α]${}_{D}^{20}$ = +3.17° (*c* = 0.3, CH_2_Cl_2_).

*Biotransformation of (***3***) by* D. igniaria *KCh6670.* Biotransformation of epoxylactone (**3**) (0.1 g), after four days, gave (+)-(**5**) (ee = 99%) as the only product. After column chromatography, 0.048 g (48% yield) of (+)-(**5**) was isolated, [α]${}_{D}^{20}$ = +1.52° (*c* = 1.5, CH_2_Cl_2_).

#### 3.5.1. Identification of Product

*5-Hydroxy-5-methyl-6-pentyltetrahydro-2H-pyran-2-one* (**4**): Colorless liquid; ^1^H-NMR (CDCl_3_), δ: 0.83 (t, 3H, *J* = 6.8 Hz, CH_3_-11), 1.19–1.44 (m, 8H, one of CH_2_-7, CH_2_-8, CH_2_-9, CH_2_-10, -OH), 1.30 (s, 3H, CH_3_-12), 1.50 (m, 1H, one of CH_2_-7), 1.86 (m, 1H, one of CH_2_-4), 2.19 (m, 1H, one of CH_2_-4), 2.57 (two d, 2H, *J* = 7.9 Hz, CH_2_-3), 3.46 (dd, 1H, *J* = 8.9 and 3.1 Hz, H-6). ^13^C-NMR (CDCl_3_), δ: 177.33 (C-2), 89.26 (C-5), 76.90 (C-6), 31.70, 30.69 (C-7, C-9), 30.64 (C-4), 29.29 (C-3), 25.92 (C-8), 22.57 (C-10), 21.26 (C-12), 14.05 (C-11). IR (film, cm^−1^): 3445 (s), 1765 (s).

*5-Methyl-6-pentyl-1,13-dioxabicyclo[4.1.0]heptan-2-ol* (**5**): Colorless liquid; ^1^H-NMR (CDCl_3_), δ: 0.89 (t, 3H, *J* = 7.0 Hz, CH_3_-11), 1.25–1.36 (m, 5H, CH_2_-10, CH_2_-9, one of CH_2_-3), 1.39 (s, 3H, CH_3_-12), 1.45 (ddd, 1H, *J* = 13.8, 6.6, 1.8 Hz, one of CH_2_-4), 1.52-1.57 (m, 2H, CH_2_-8), 1.61 (s, 1H, -OH), 1.68 (ddd, 1H, *J* = 13.8, 9.6, 5.4 Hz, one of CH_2_-4), 1.86–1.89 (m, 2H, CH_2_-7), 2.09 (ddd, 1H, *J* = 14.4, 9.6, 6.6, one of CH_2_-3), 4.31 (d, 1H, *J* = 6.0 Hz, H-2). ^13^C-NMR (CDCl_3_), δ: 73.52 (C-2), 71.49 (C-5), 68.09 (C-6), 32.26 (C-9), 30.53 (C-7), 29.79 (C-4), 25.50 (C-3), 25.04 (C-8), 22.65 (C-10), 15.46 (C-12), 14.05 (C-11). IR (film, cm^−1^): 3430 (s), 1457 (m).

#### 3.5.2. Feeding Deterrent Activity—Bioassays

*Insect cultures.* In these studies, a laboratory-reared strain of the lesser mealworm collected from poultry house litter in a local, commercial broiler farm located near Toruń (Poland) was used. The colony was kept in glass containers in a rearing chamber at +29 °C in the dark. As a food medium, a mixture consisting of one part of oat flakes, one part of wheat bran, and 0.01 part of brewers’ yeast was applied. To maintain moisture levels at ca. 55%, fresh apple halves were placed in the containers. Using the culture method of Rice and Lambkin [33], we obtained large numbers of insects of approximately the same age for the tests.

The stock culture of the peach potato aphid was maintained as a multiclonal colony on Chinese cabbage *Brassica rapa* L. ssp. *pekinensis* L. under laboratory conditions at 65% R.H., 20 °C, and long day photoperiod (L16:8D) in a growing chamber Sanyo MLR-351H (Sanyo Electronics Co. Ltd., Moriguchi, Japan). To maintain colony vitality, apterous aphids were transferred to non-infested plants every other week. For all experiments involving aphids, one to seven days old adult apterous females of *M. persicae* were collected from the stock culture.

*Feeding deterrent acti*v*ity tests.* The feeding deterrent activities of compounds studied against larvae and adults of *A. diaperinus* were determined by using the standard method of choice and no-choice tests as previously described by Gliszczyńska et al. [22]. For the feeding assays, acetone solutions of the test compounds at a concentration of 10 mg·mL^−1^ were prepared. A dose of 1 mL of solution or acetone alone as control was applied to 1 g of oat flakes (Melvit, Warsaw, Poland) by micropipette. After evaporation of the solvent (30 min of air drying), the flakes were weighed and offered to 10 larvae (25–30 days old) or 10 unsexed adults (7–10 days old) during the following three-day period. In the choice test, both control and treated oat flakes were placed together in a Petri dish (15 cm diameter), with the control flakes separated from the treated flakes by a thin glass capillary. In the no-choice test, insects were exposed to only one kind of food—treated or control. Dishes were kept in the rearing chamber at 29 ± 1 °C in the dark. After three days of feeding and the re-weighing of the remaining flakes, the relative (R) and absolute (A) deterrence coefficients were calculated using the following formula:

R = (C − E)/(C + E) × 100

where C and E are the weights of the control and treated foods consumed by the insects in the choice test, respectively. The absolute deterrence coefficient A was calculated using the same formula, but C and E were obtained from no-choice test. The measure of the deterrent activity of tested compounds is the total coefficient of deterrence: *T* = *A* + *R*. The total coefficient of deterrence, which ranged from −200 to 200, served as the index activity. The compounds with *T* values ranging from 151 to 200 are very good deterrents, those with *T* values lower than 50 are weak deterrents. The remaining values indicate good (101–150) or moderate (51–100) antifeedant activity. Negative *T* values point to attractant properties of the compound.

Aphid responses to the compounds studied were measured as a settling success in the choice situation and initial aphid responses in no-choice test, as described previously [22]. The compounds studied were applied as 0.1% solutions in 70% ethanol to the test leaves of Chinese cabbage that were detached from plants grown in the laboratory at 65% R.H., 20 °C, and long-day photoperiod (L16:8D) in a growing chamber Sanyo MLR-351H (Sanyo Electronics Co. Ltd.). For uniform distribution of the compound, the leaves were immersed in the studied solutions for 20 s. Control leaves were immersed in 70% ethanol. For aphid settling bioassay, control and treated leaves were placed in the Petri dish and the test aphids were placed in an equal distance from either leaf. The leaves were offered to aphids 1 h after the application of the studied solutions to allow the evaporation of the solvent. Aphids that settled on each leaf were counted 1, 2, and 24 h after having access to the leaves (8 replicates, 20 adult apterous aphids/replicate). Aphids that did not settle on any of the leaves were discarded from calculations. The results were statistically analyzed using Student *t*-test: the number of aphids on control leaves was compared to the number of aphids on the test leaf for each compound/time interval separately. In the initial response bioassay, aphid behaviour was studied by direct observation of the freely moving individuals on a leaf treated with the tested compounds, using a video camera. The experiment was carried out for 15 min (16 aphids/compound). The duration of probing was recorded basing on the relationship between antennal and body movements and penetration of the stylets as described by Hardie et al. [34]. The position of antennae parallel to the abdomen and the cessation of body movements were associated with stylet penetration. The total time spent by aphids on leaves, total probing time, number of probes, and mean probing time were determined from this experiment. The data were analysed using one-way ANOVA followed by the Tukey test.

## 4. Conclusions

In conclusion, we introduced chemical Baeyer–Villiger oxidation and an enantioselective fungi-mediated conversion to produce novel alkyl-substituted δ-lactones from dihydrojasmone (**1**). The experiments confirmed that the lactone moiety and additional oxygen function introduced into the structure of dihydrojasmone (**1**) significantly increased and altered the quality of its activity. The activities of dihydrojasmone (**1**) and its derivatives (**2**–**5**) were species- and developmental stage-specific. The most potent antifeedant activity against *A. diaperinus* was determined for δ-hydroxylactone (−)-(**4**), which strongly inhibited the feeding of larvae and adults. The saturated bicyclic δ-lactone (**3**) expressed clear attractant properties towards *M. persicae*.

## Figures and Tables

**Table 1:** The reaction rates of production of hydroxylactone (**4**) by selected microorganisms.

Entry	Microorganism	Transformation Period (Days)	Reaction Rate (mg/h) of Product
1	*F. culmorum* AM10	1	4.1 × 10^−1^
		2	8.3 × 10^−3^
		4	Full conversion
		6	Full conversion
		9	Full conversion
2	*F. equiseti* AM15	1	2.2 × 10^−1^
		2	9.2 × 10^−2^
		4	3.9 × 10^−2^
		6	1.5 × 10^−2^
		9	Full conversion
3	*B. bassiana* AM278	1	1.6 × 10^−1^
		2	7.1 × 10^−2^
		4	6.5 × 10^−2^
		6	2.9 × 10^−2^
		9	Full conversion

**Table 2:** Feeding deterrent activity of the studied compounds in choice and no-choice tests against *A. diaperinus*.

Compound	Deterrence Coefficients ± SE ^a^					
	Larvae			Adults		
	A	R	T	A	R	T
(**1**)	29.37 ± 0.91bc	71.40 ± 4.17bc	100.77 ± 3.70b	−20.69 ± 7.61a	91.48 ± 1.02b	70.79 ± 8.04a
(**2**)	40.88 ± 3.77c	88.84 ± 0.65c	129.72 ± 3.97bc	57.17 ± 14.42b	93.19 ± 0.51b	150.36 ± 11.87b
(−)-(**4**)	74.95 ± 3.57d	78.29 ± 3.59c	153.24 ± 7.09c	90.21 ± 0.3b	72.26 ± 7.51a	162.46 ± 7.69b
(+)-(**4**)	−8.99 ± 2.67a	53.21 ± 9.14ab	44.22 ± 8.36a	82.69 ± 3.27b	97.02 ± 0.49b	179.71 ± 2.81b
(**3**)	8.35 ± 2.85ab	47.39 ± 4.64a	55.74 ± 7.34a	−28.64 ± 9.50a	64.48 ± 2.31a	35.84 ± 8.38a
(+)-(**5**)	4.93 ± 2.09ab	90.05 ± 4.09c	94.98 ± 14.68b	nt	nt	nt

^a^ Values are the means of the four replicates (±SE = Standard Error), each set up with ten larvae or adults (*n* = 10). A: absolute coefficients; R: relative coefficients; T: total coefficients. nt: Not tested. Means followed by the same letters within each column are not significantly different (one-way ANOVA and Tukey’s test (*p* < 0.05).

**Table 3:** Settling success of *Myzus persicae* on leaves treated with dihydrojasmone (**1**) and alkyl-substituted δ-lactones (**2**), (**3**), and (−)-(**4**).

Compound	Number of Aphids ± SE ^a^					
	1 h		2 h		24 h	
	C	T	C	T	C	T
(**1**)	7.9 ± 0.8	6.0 ± 0.9	7.8 ± 0.9	8.0 ± 0.8	7.9 ± 0.8	7.5 ± 0.6
(**2**)	8.1 ± 1.1	7.0 ± 1.0	7.6 ± 1.1	6.1 ± 1.3	8.4 ± 1.3	6.5 ± 1.1
(−)-(**4**)	5.8 ± 0.7	7.5 ± 0.5	6.4 ± 1.0	7.5 ± 0.7	7.8 ± 0.7	7.6 ± 0.6
(**3**)	5.5 ± 1.0	10.5 ± 0.9 *	6.4 ± 0.9	10.6 ± 0.6 *	7.0 ± 0.8	11.0 ± 0.7 *

^a^ Values are means of the eight replicates (±SE = Standard Error), each set up with 20 adult apterae (*n* = 20). * Asterisks indicate statistically significant differences between the number of aphids settled on control leaves (C) and leaves with a tested compound (T) at 1, 2, and 24 h intervals (Student *t*-test; *p* < 0.05).

**Table 4:** Immediate behavioural responses of *Myzus persicae* during initial 15 min. contact with plants treated with dihydrojasmone (**1**) and alkyl-substituted δ-lactones (**2**), (**3**), and (−)-(**4**).

	Compounds				
	Control	(1)	(2)	(−)-(4)	(3)
Time to 1st probe (s)	25.6 (±8.5)	50.1 (±18.8)	21.3 (±7.1)	13.6 (±4.3)	67.9 (±37.0)
Total time on leaf (s)	900.0 (±0.0)	644.1 (±118.8) *	900.0 (±0.0)	900.0 (±0.0)	757.7 (±77.2) *
Total time out of the leaf (s)	0.0 (±0.0)	255.9 (±118.8) *	0.0 (±0.0)	0.0 (±0.0)	142.3 (±77.2) *
Total probing time (s)	735.3 (±47.4)	443.3 (±110.0) *	789.2 (±32.9)	748.7 (±29.2)	626.6 (±122.4)
Number of probes	4.3 (±0.9)	2.8 (±0.8)	2.6 (±0.5)	3.7 (±0.4)	1.6 (±0.3) *
Mean probing time (s)	255.2 (±53.7)	236.7 (±91.7)	464.6 (±108.7)	268.2 (±75.7)	398.6 (±104.2)

Values represent means ± SE (= Standard Error). Time given in seconds, * asterisks indicate statistically significant differences in relation to control at *p* ≤ 0.05.

## Supplementary Materials

Supplementary materials can be accessed at: http://www.mdpi.com/1420-3049/21/9/1226/s1.
