# Supplementary material for: Alkyl-Substituted δ-Lactones Derived from Dihydrojasmone and Their Stereoselective Fungi-Mediated Conversion: Production of New Antifeedant Agents
Source: Molecules. 2016 Sep 13;21(9):1226. doi: 10.3390/molecules21091226 (PMC6273879; doi:10.3390/molecules21091226)
Supplement: Supplementary file 1 [file molecules-21-01226-s001.pdf]

Supplementary Materials: Alkyl-Substituted δ-Lactones Derived from Dihydrojasmone and Their Stereoselective Fungi-Mediated Conversion: Production of New Antifeedant Agents

Anna Gliszczyńska, Damian Semba, Maryla Szczepanik, Katarzyna Dancewicz and Beata Gabryś

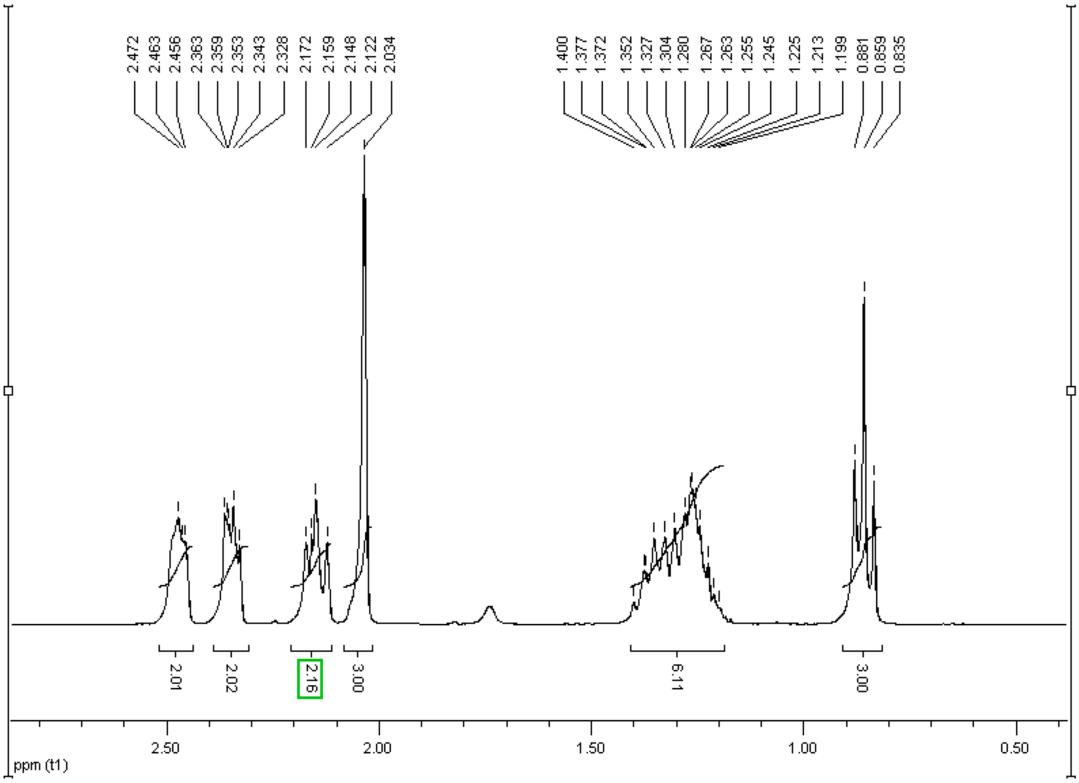

Figure S1. ^{1}H NMR spectrum of **1**.

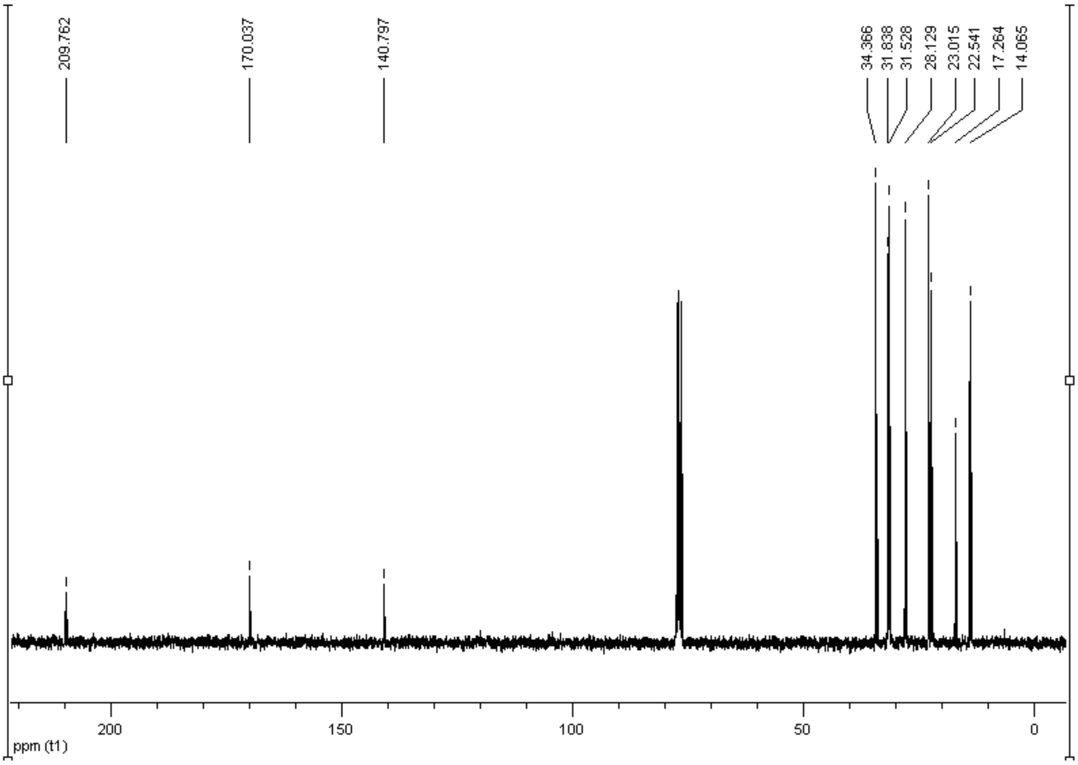

Figure S2. ^{13}C NMR spectrum of **1**.

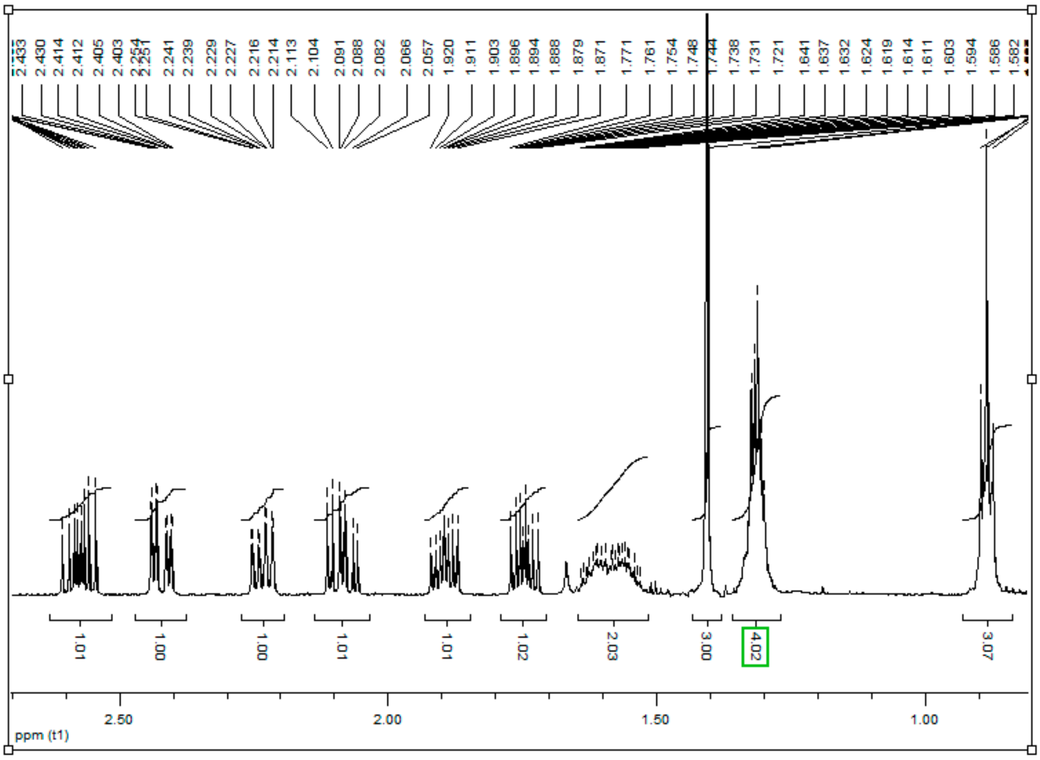

Figure S3. ^{1}H NMR spectrum of **2**.

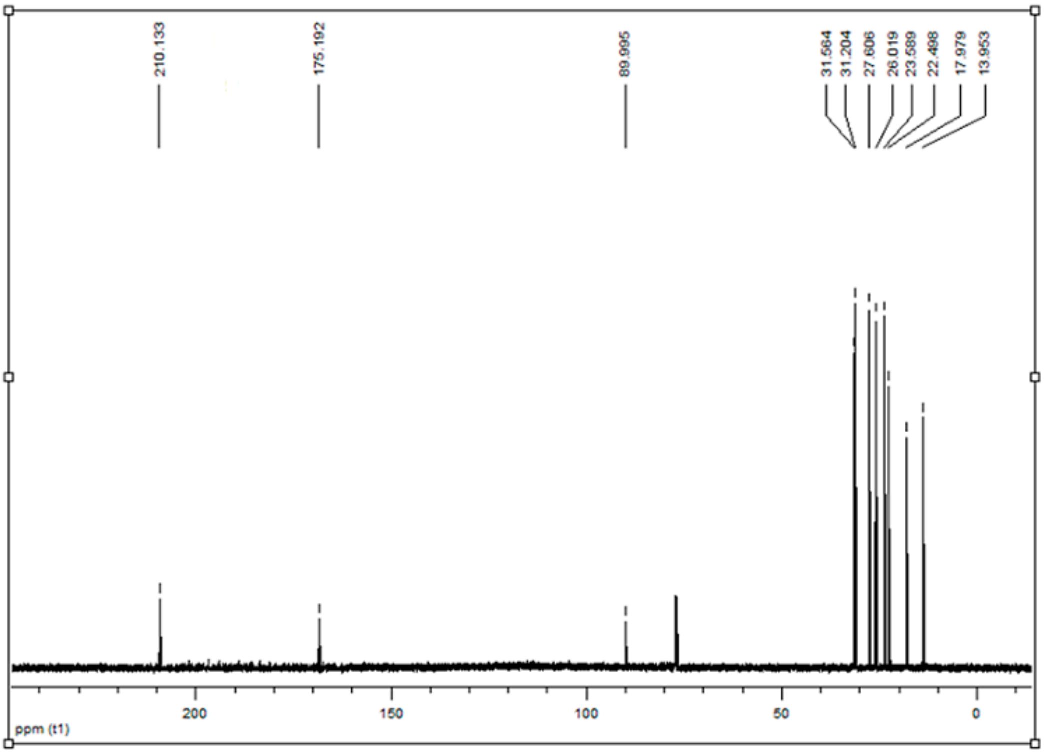

Figure S4. ^{13}C NMR spectrum of **2**.

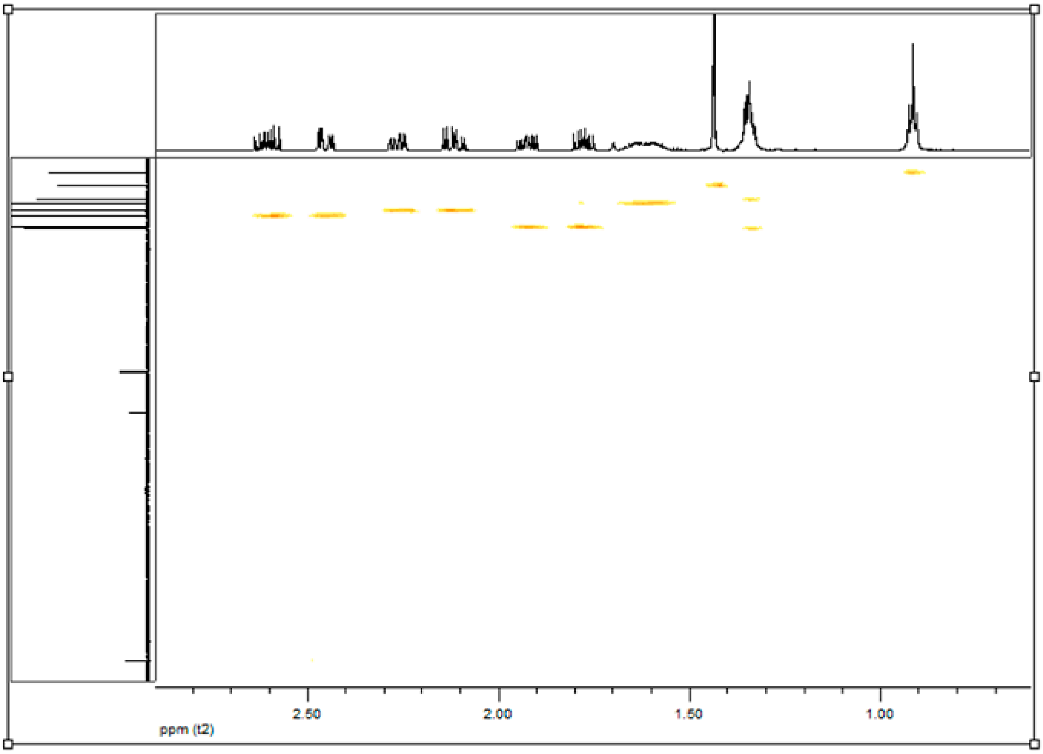

Figure S5. HSQC spectrum of **2**.

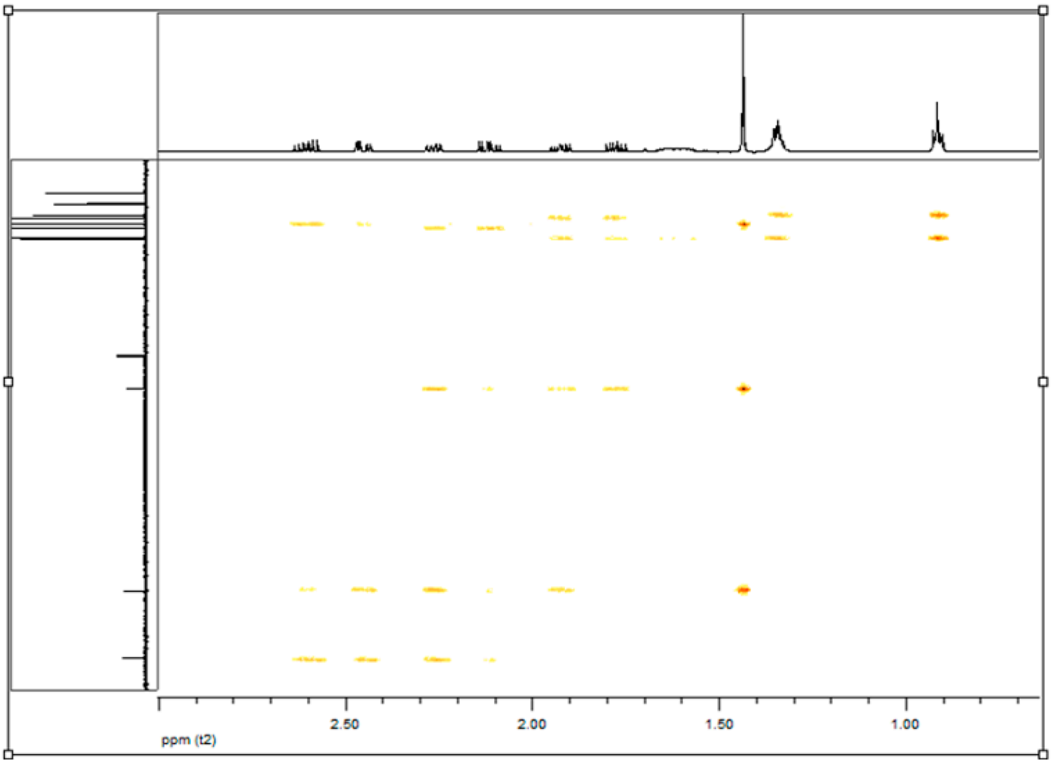

Figure S6. HMBC spectrum of **2**.

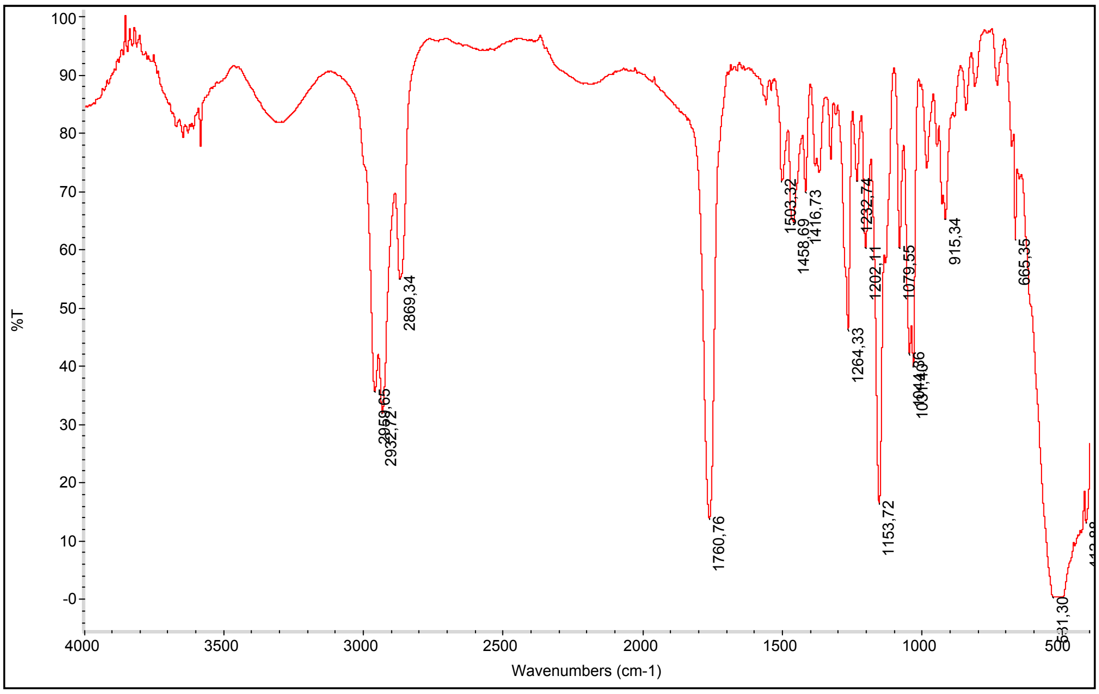

Figure S7. IR spectrum of **2**.

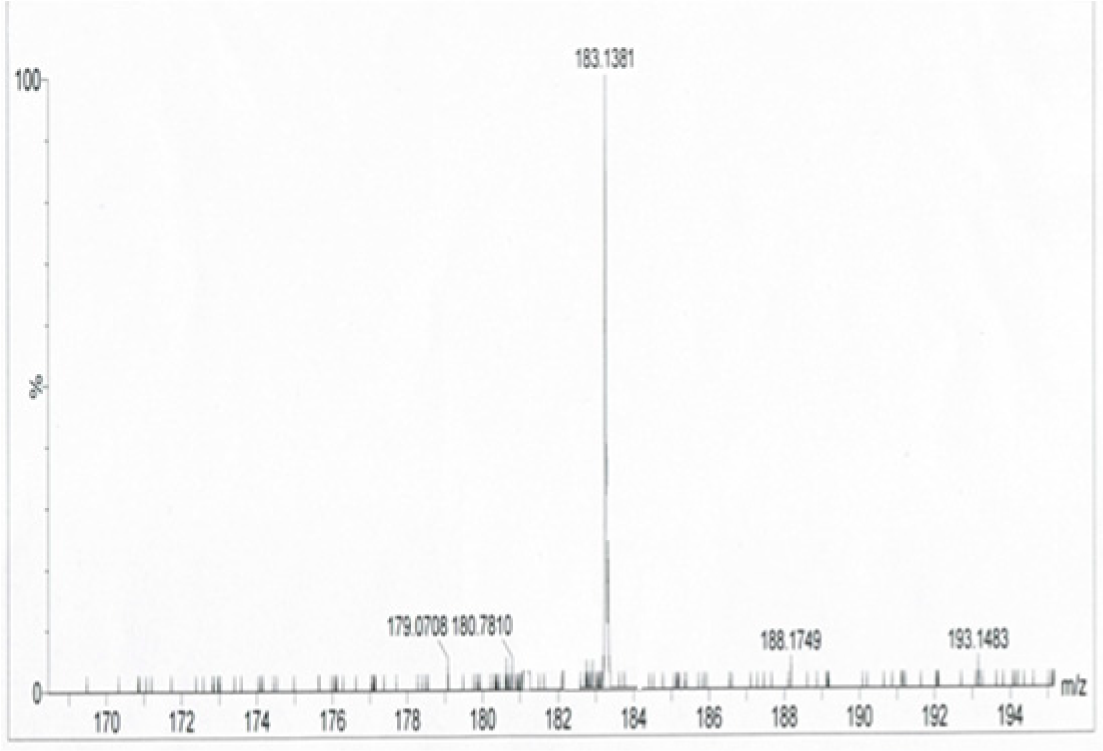

Figure S8. Mass spectrum of **2**.

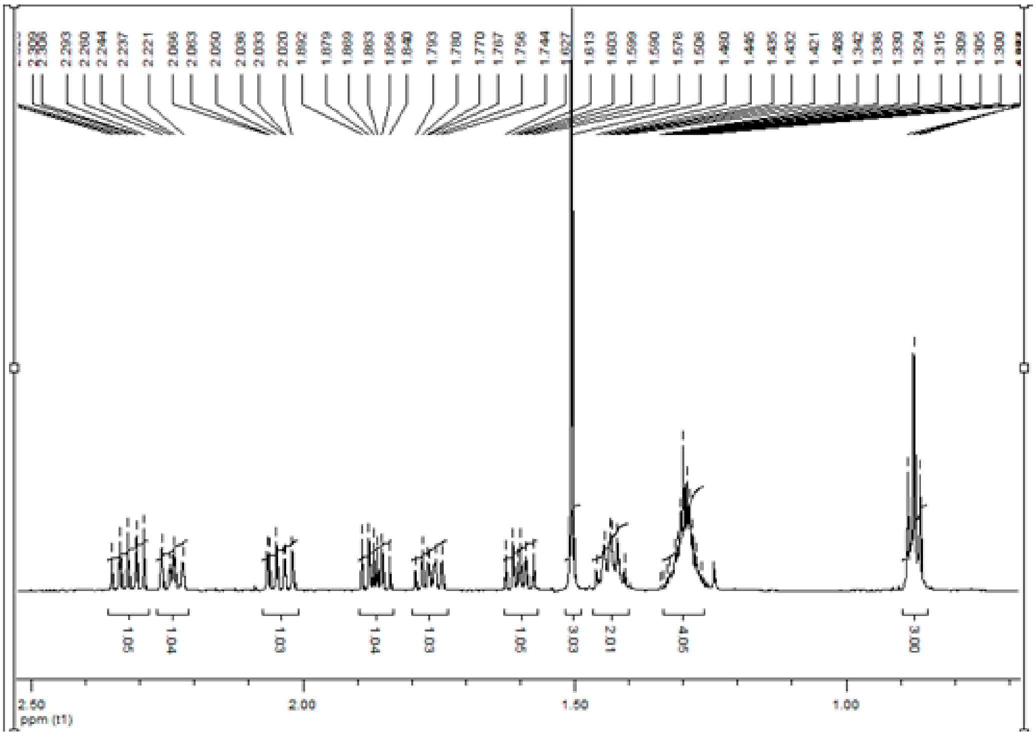

Figure S9. Figure ^{1}H NMR spectrum of **3**.

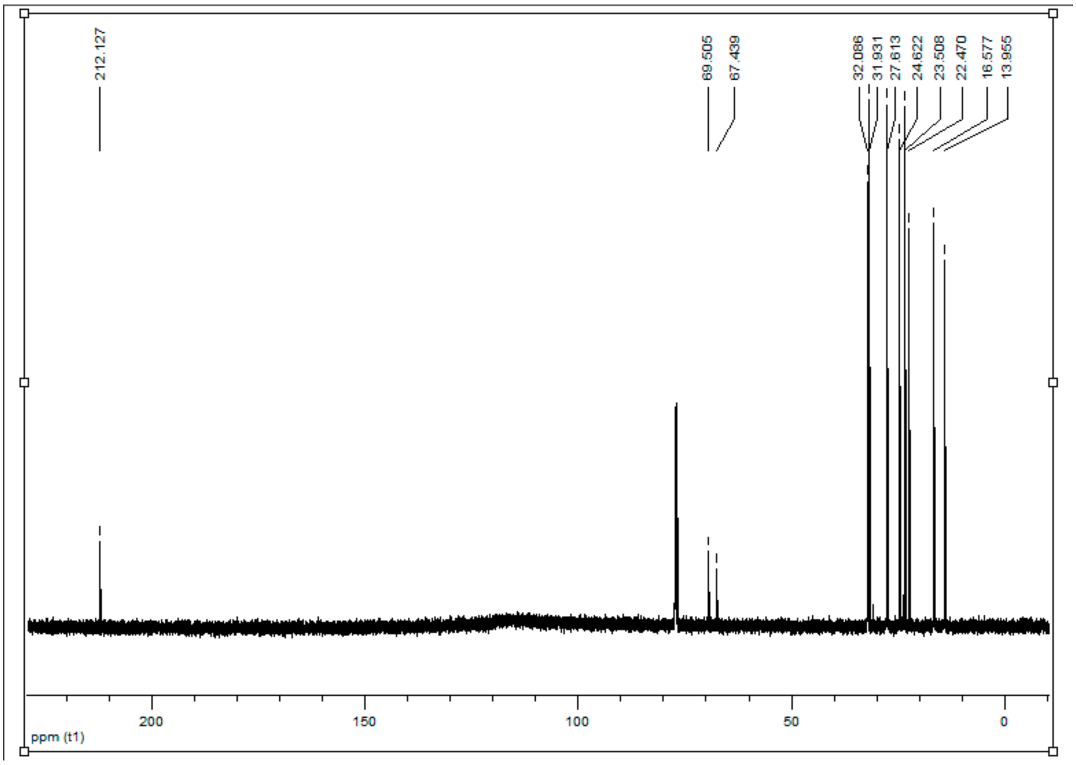

Figure S10. 13C NMR spectrum of **3**.

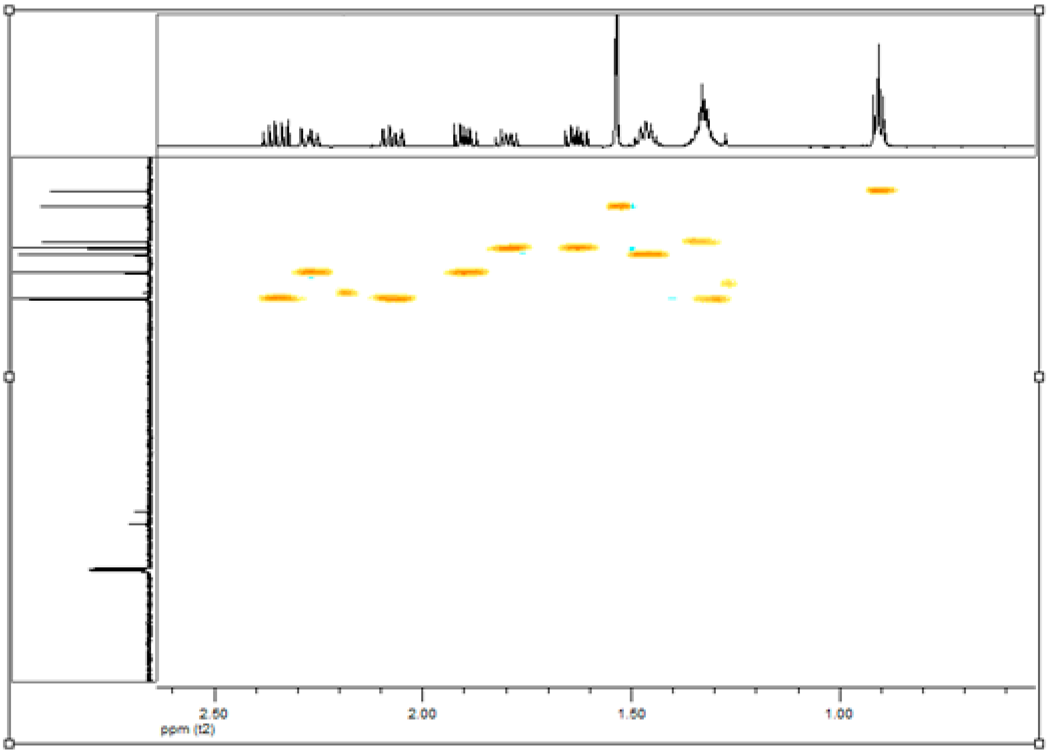

Figure S11. HSQC spectrum of **3**.

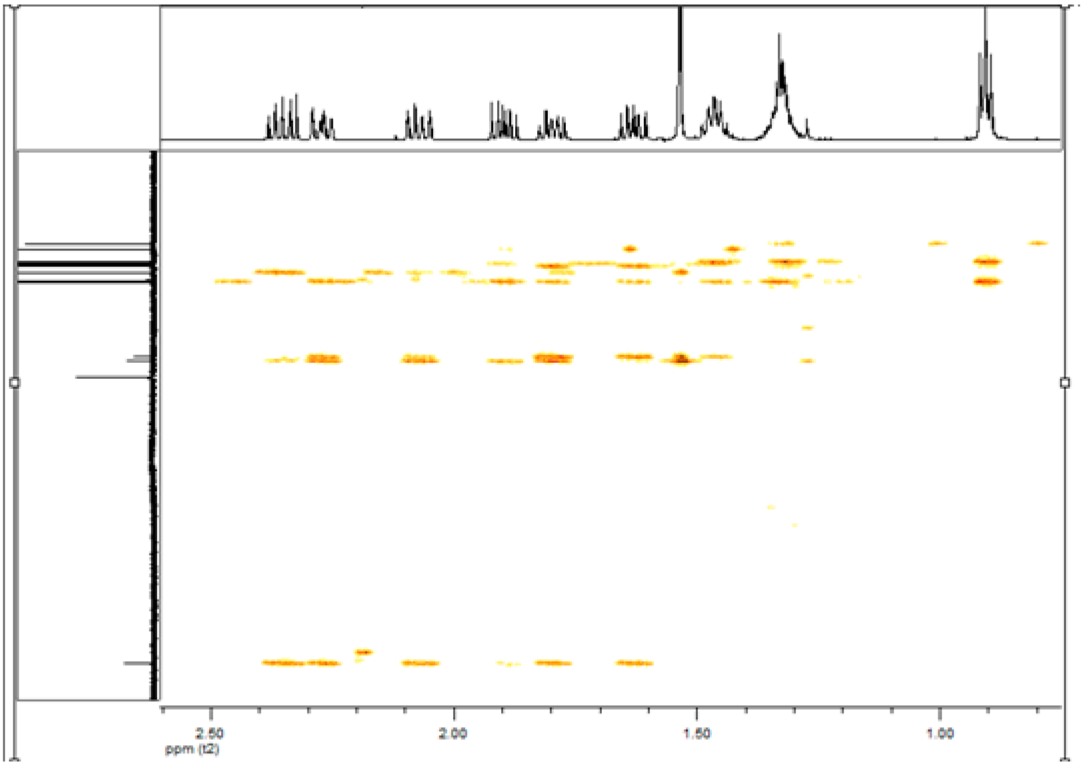

Figure S12. HMBC spectrum of **3**.

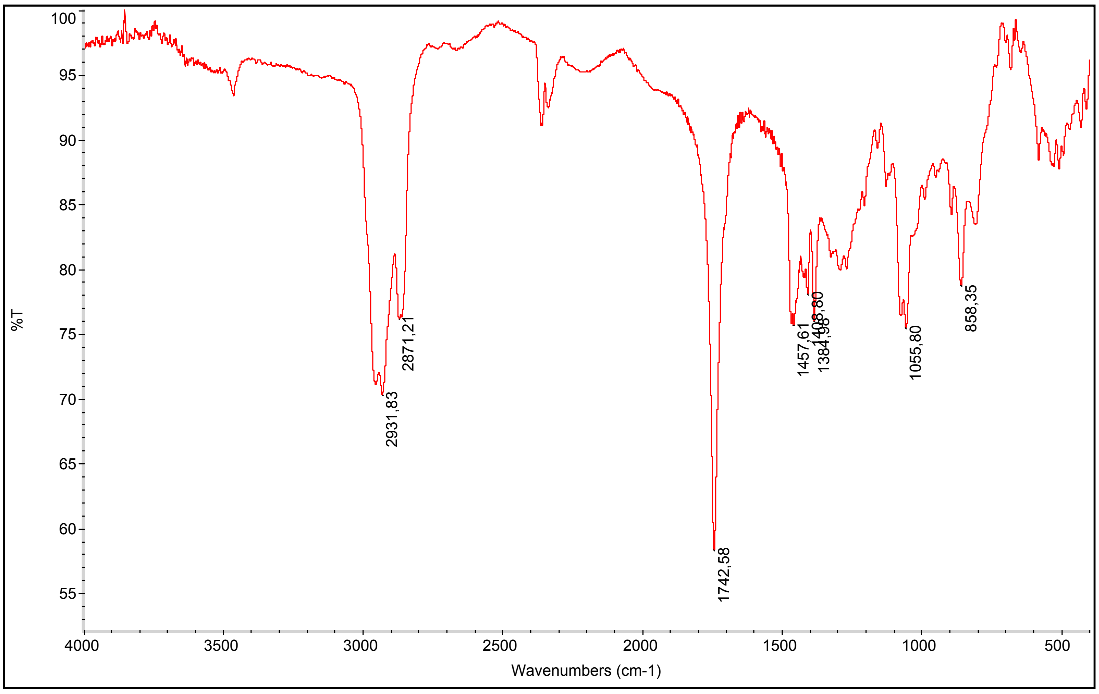

Figure S13. IR spectrum of **3**.

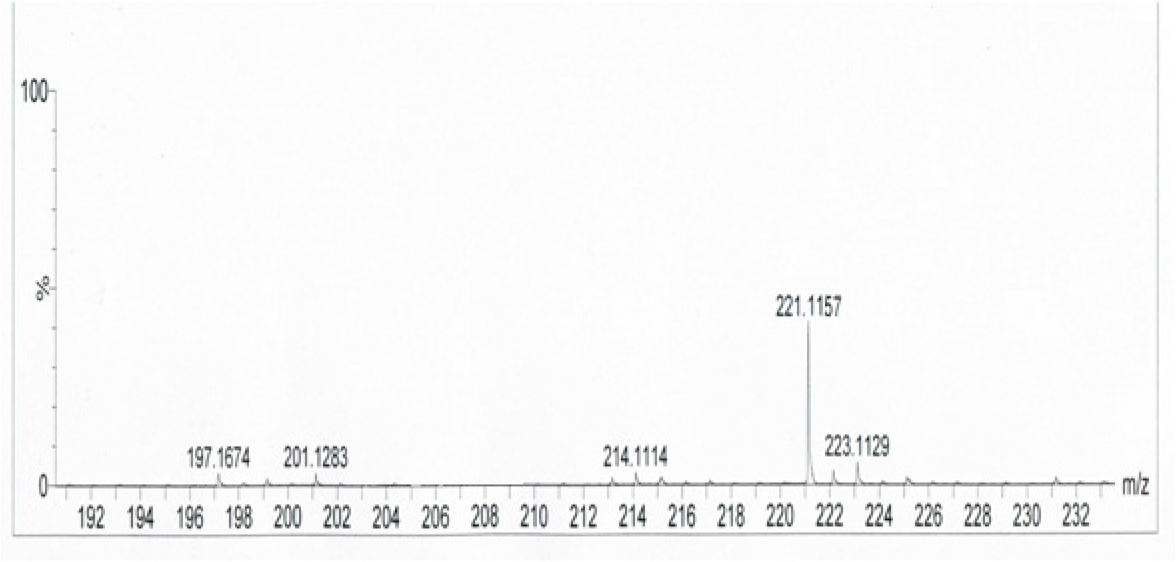

Figure S14. Mass spectrum of **3**.

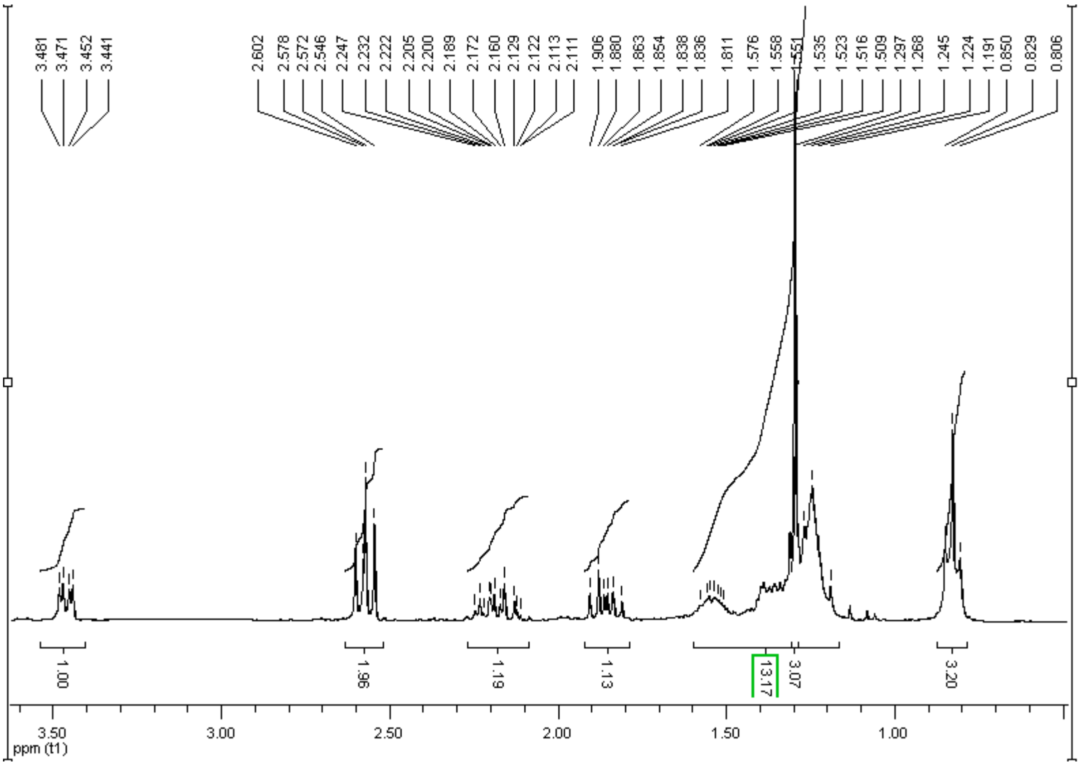

Figure S15. ^{1}H NMR spectrum of **4**.

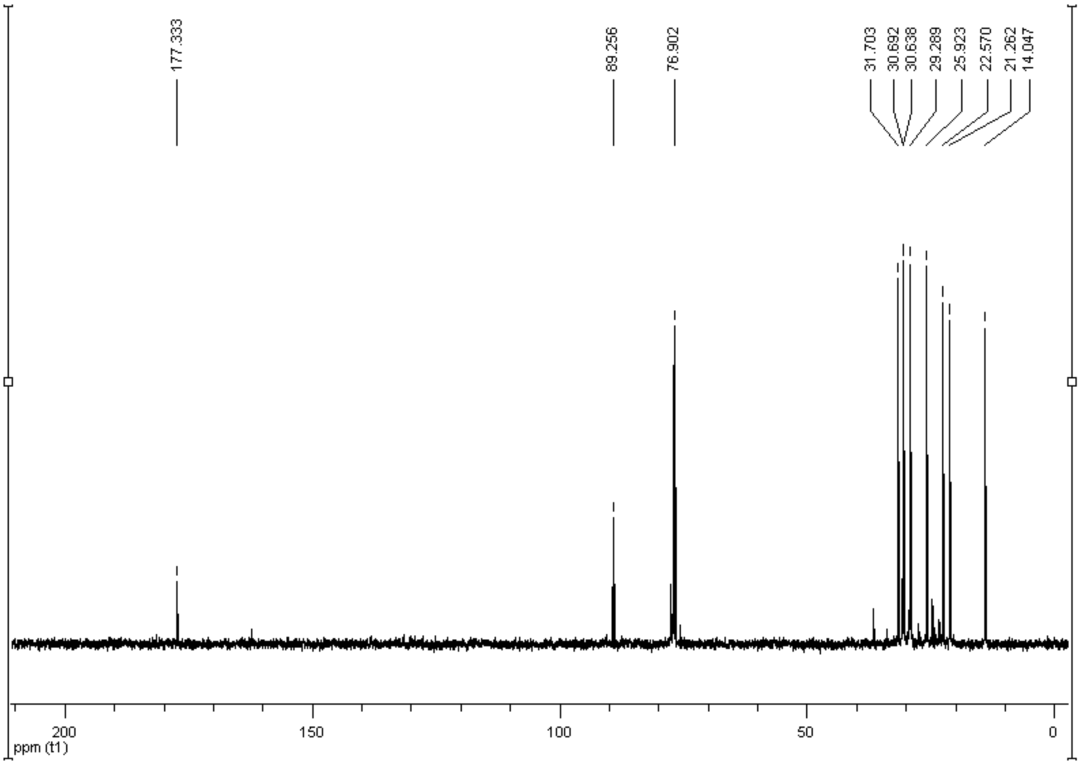

Figure S16. ^{13}C NMR spectrum of **4**.

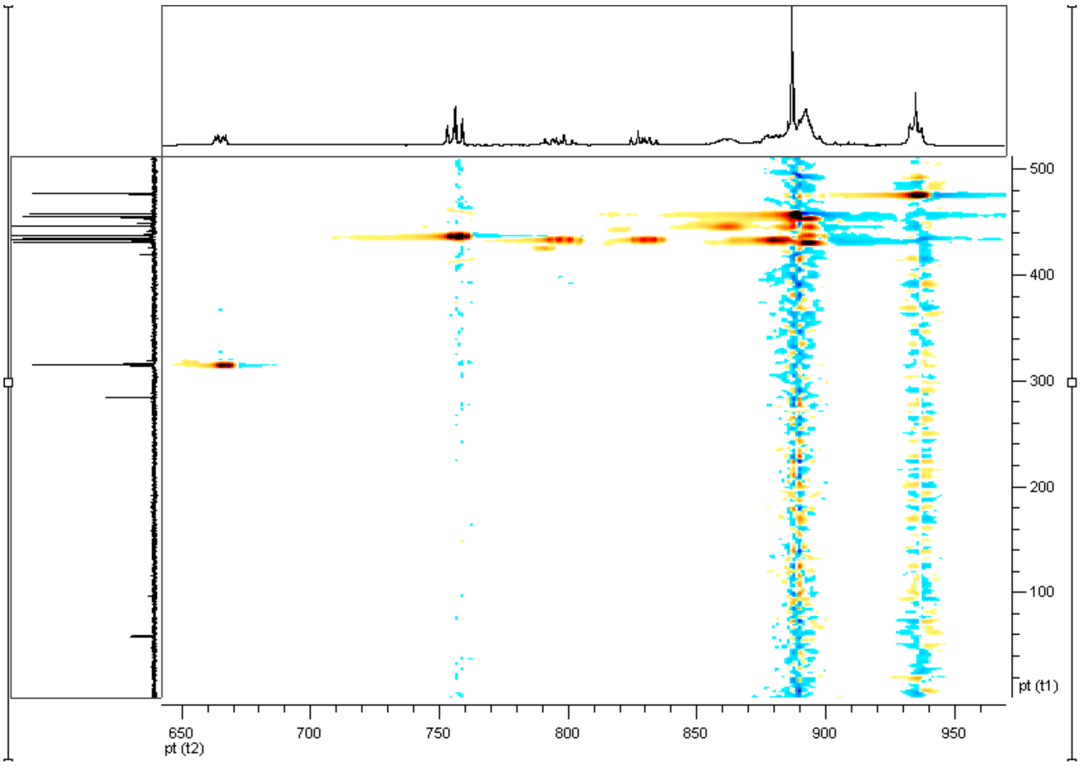

Figure S17. HSQC spectrum of **4**.

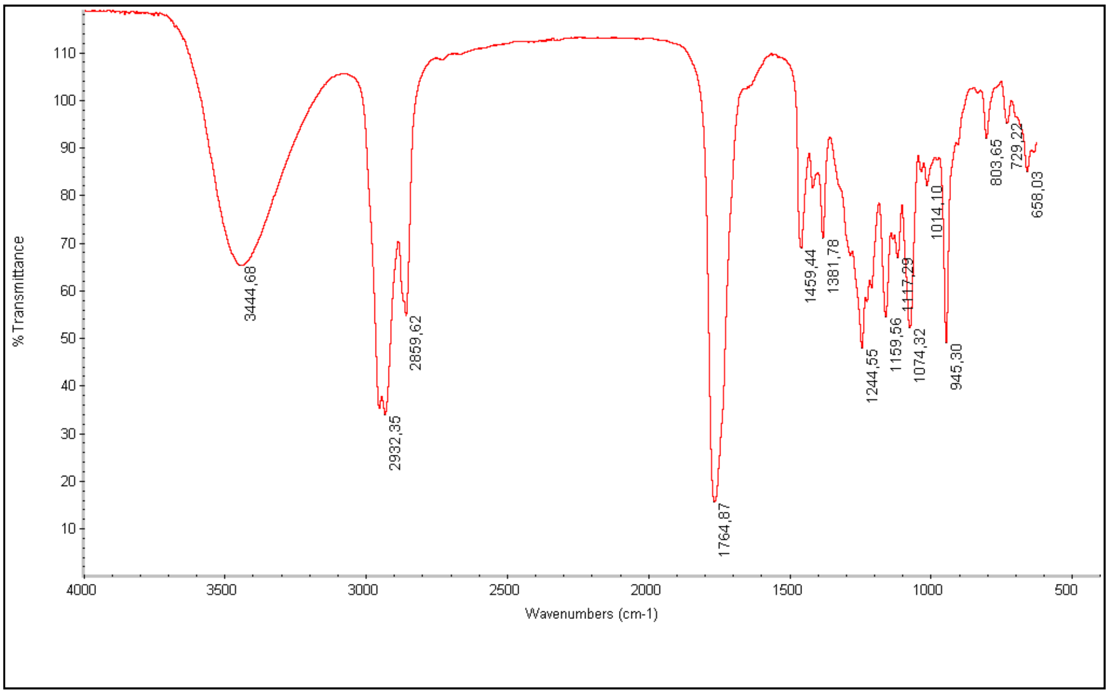

Figure S18. IR spectrum of **4**.

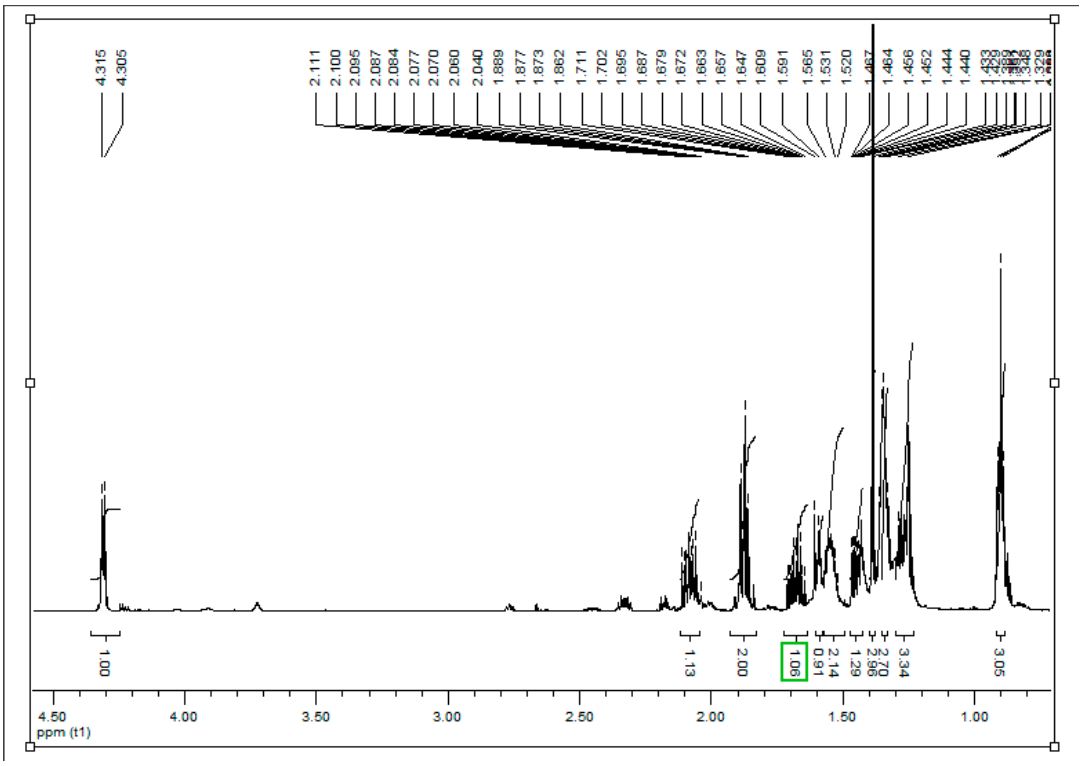

Figure S19. 1H NMR spectrum of **5**.

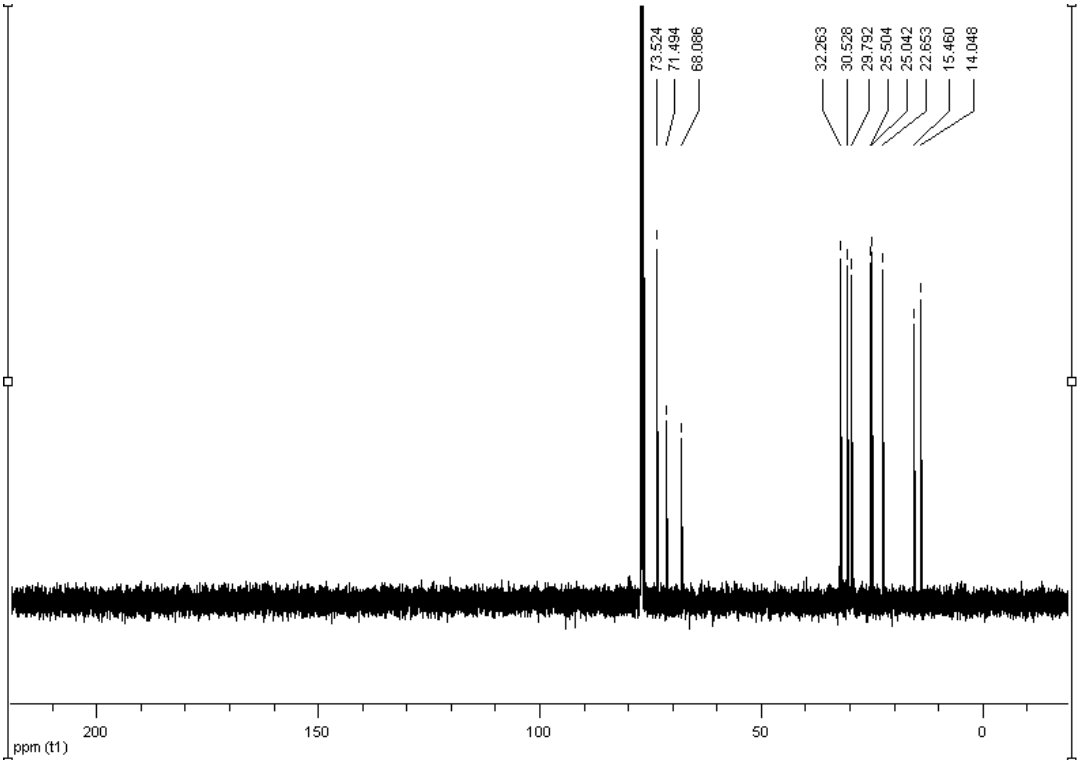

Figure S20. ^{13}C NMR spectrum of **5**.

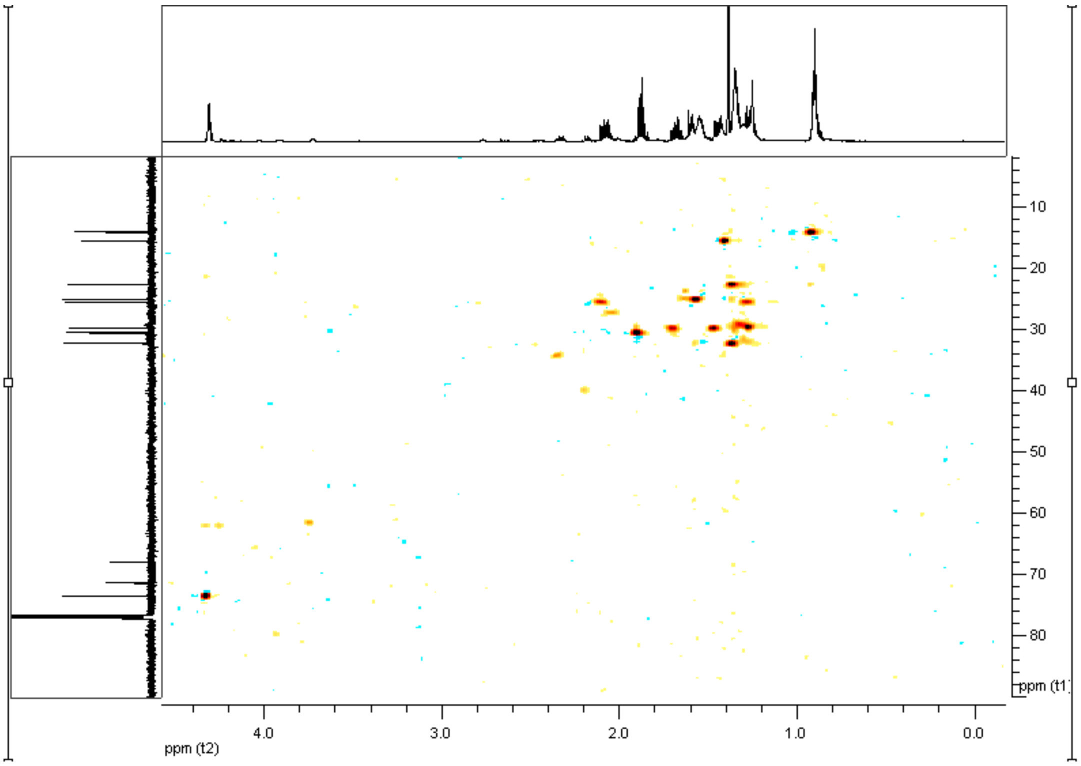

Figure S21. HSQC spectrum of **5**.

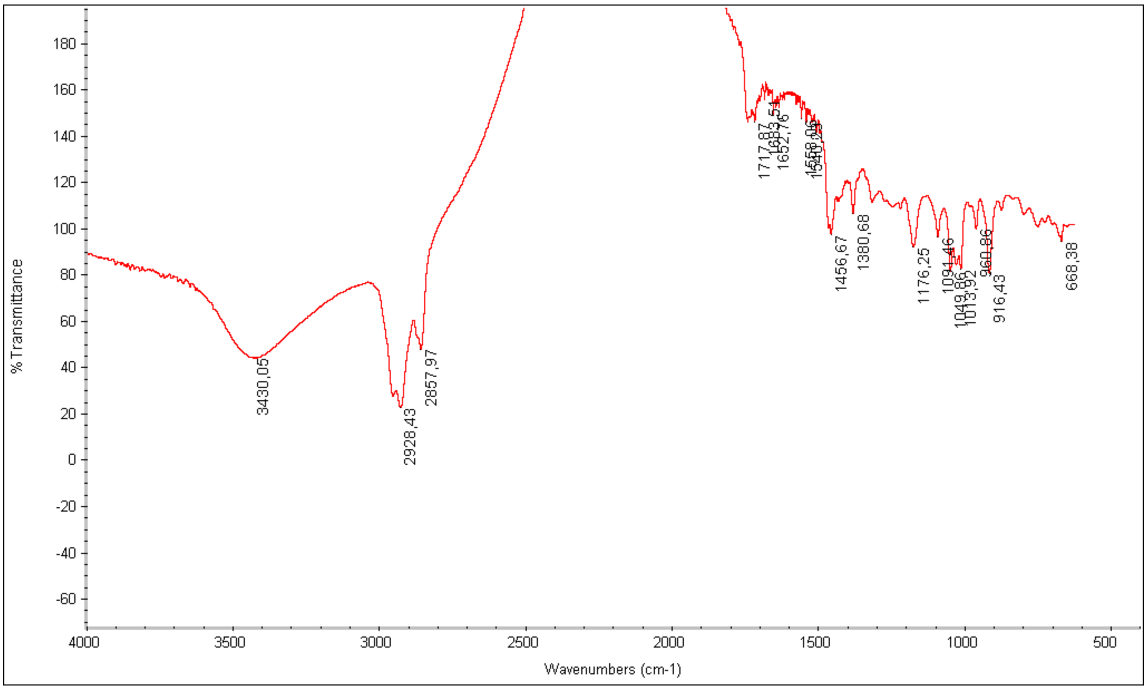

Figure S22. IR spectrum of **5**.

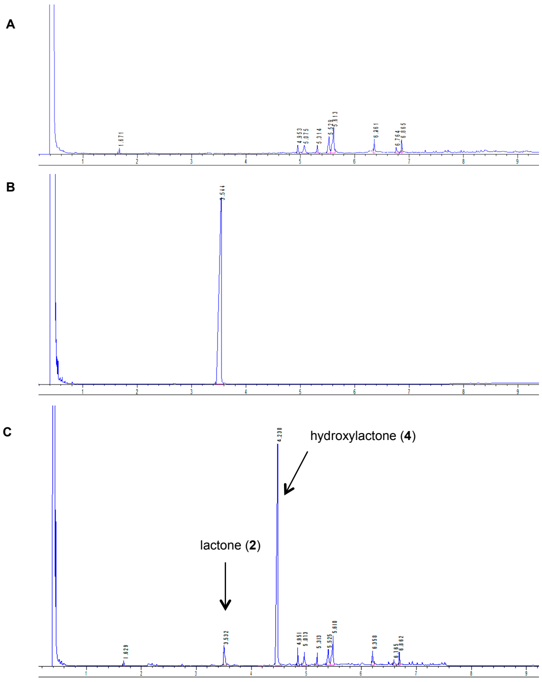

Figure S23. GC chromatograms obtained during the experiment with *F. culmorum* AM10: metabolites of *F. culmorum* AM10 (**A**), substrate of biotransformation lactone (**2**) (**B**), the result of biotransformation of lactone (**2**) after 1 day (**C**).
